# Development of a machine learning predictive model for early detection of breast cancer

**DOI:** 10.12688/f1000research.161073.1

**Published:** 2025-02-05

**Authors:** Rinsy Rahman, Dola Saha, Winniecia Dkhar, Sathyendranath Malli, Neil Barnes Abraham

**Affiliations:** 1 Department of Health Information Management, Manipal College of Health Professions, Manipal Academy of Higher Education, Manipal, Karnataka, 576104, India; 2 Department of Medical Imaging Technology, Manipal College of Health Professions, Manipal Academy of Higher Education, Manipal, Karnataka, 576104, India; 3 School of Information Science, Manipal Academy of Higher Education, Manipal, Karnataka, 576104, India

**Keywords:** Breast cancer, Mammography, Machine learning, Tumor classification, Predictive modelling

## Abstract

**Background:**

Breast cancer remains a significant global health concern, with over 7.8 million cases reported in the last five years. Early detection and accurate classification are crucial for reducing mortality rates and improving outcomes. Machine learning (ML) has emerged as a transformative tool in medical imaging, enabling more efficient and accurate diagnostic processes.

**Objective:**

This study aims to develop a machine learning-based predictive model for early detection and classification of breast cancer using the Wisconsin Breast Cancer Diagnostic dataset.

**Methods:**

The dataset, comprising 569 samples and 32 features derived from fine needle aspirate biopsy images, was pre-processed through data cleaning, normalization using the Robust Scaler, and feature selection. Five supervised ML algorithms—Logistic Regression, Support Vector Classification (SVC) with linear and radial basis function (RBF) kernels, Decision Tree, and Random Forest—were implemented. Models were evaluated using performance metrics, including accuracy, precision, sensitivity, specificity, and F1 scores.

**Results:**

The SVC-RBF model demonstrated the highest accuracy (98.68%) and balanced performance across other metrics, making it the most effective classifier for distinguishing between benign and malignant tumors. Key features such as texture mean and area (worst) significantly contributed to classification accuracy.

**Conclusions:**

This study highlights the potential of ML algorithms, particularly SVC-RBF, to revolutionize breast cancer diagnostics through improved accuracy and efficiency. Future research should validate these findings with diverse datasets and explore their integration into clinical workflows to enhance decision-making and patient care.

## 1. Introduction

Breast cancer is a global health concern that affects millions of women worldwide. The alarming number of diagnoses highlights the importance of proactive measures such as regular screenings, self-examination, and increased awareness. In the last five years alone, a staggering 7.8 million women have been diagnosed with this disease. ^1^ These numbers underscore the urgent need for increased awareness, early detection, and effective treatment options. The health system must be significantly reinforced to enhance breast cancer outcomes. In order to reduce mortality rates and provide effective treatment, early detection and screening of breast cancer are highly important. ^2, 3^ Early detection is therefore essential to ensure the best outcome in treating breast cancer. It is well known that rapid diagnosis with machine learning is highly beneficial considering the rise in breast cancer cases. ^4^

The integration of AI in breast cancer detection and diagnosis has the potential to revolutionize the field of oncology. ^5, 6^ In recent years, machine learning (ML) algorithms have emerged as powerful tools in the field of medical imaging, offering the potential to enhance the accuracy and efficiency of tumour detection and classification. ^7, 8^ Machine learning algorithms can analyse vast amounts of data and identify patterns that may not be apparent to human experts. Machine learning algorithms can be trained to analyse mammograms and provide additional insights to radiologists, helping them make more informed decisions. It is imperative that healthcare providers and researchers continue to explore and harness the power of AI to further enhance breast cancer care. ^8– 10^

The aim of this study is to develop a machine learning predictive model specifically designed for early detection and classification of breast cancer. By leveraging ML algorithms, the goal is to improve the accuracy of tumour detection and significantly reduce the time required for cell identification.

## 2. Methods

### 2.1 Study design and setting

This research was conducted within the Health Informatics Laboratory, Department of Health Information Management, Manipal College of Health Professions, Manipal Academy of Higher Education, Manipal, over six months (January–June 2022). The study aimed to develop and evaluate a machine learning predictive model for early detection and differential diagnosis of benign and malignant breast lesions.

### 2.2 Data source and inclusion criteria

The Wisconsin Breast Cancer Diagnostic dataset, available on Kaggle, ^11^ was utilized. This dataset comprises 569 records and 33 features derived from fine needle aspirate (FNA) biopsy images, representing tumor characteristics. Data of female patients aged 18–70 years were included. Key features analysed included tumor radius, texture, perimeter, area, smoothness, compactness, concavity, symmetry, and fractal dimension.

### 2.3 Data preprocessing

Data preprocessing involved removing missing and null values, followed by normalization using the Robust Scaler method to mitigate outlier effects. Exploratory Data Analysis (EDA) was conducted using Python to visualize data distributions and relationships through violin plots, box plots, and correlation matrices, enabling the selection of significant features for model training.

### 2.4 Model development

Five supervised machine learning algorithms were implemented: Logistic Regression, Support Vector Classification (SVC) with linear and radial basis function (RBF) kernels, Decision Tree, and Random Forest. The dataset was split (60:40) into training and testing subsets using Scikit-learn’s train test split Models were trained on the training set and optimized using hyperparameter tuning.

### 2.5 Performance evaluation

Model performance was assessed using confusion matrices and metrics, including accuracy, precision, sensitivity (recall), specificity, and F1 scores, calculated using Scikit-learn’s classification report function. Among the models, SVC-RBF demonstrated the highest accuracy (99%), proving its efficacy for early detection and differential diagnosis of breast lesions.

### 2.6 Statistical tools and software

All analyses were performed using Python 3.8 ^12^ in Jupyter Notebook. Libraries used included Pandas (v1.2.4) ^13^ for data manipulation, Numpy (v1.20.3) ^14^ for numerical computations, Matplotlib (v3.4.2) ^15^ and Seaborn (v0.11.1) ^16^ for data visualization, and Scikit-learn (v0.24.2) ^17^ for machine learning.

### 2.7 Ethical considerations

The dataset was extracted from the online open-source Wisconsin (Diagnostics) dataset. The study approval was obtained from Institutional Research Committee of Manipal College of Health Professions, Manipal on the 20 ^th^ of January 2022 (MCHP/Mpl/IRC/PG/2022/04). All procedures adhered to established ethical guidelines for secondary data analysis and data use policies. Consent is not applicable since the data was an extracted from the online open source Wisconsin (Diagnostics) dataset.

## 3. Results

### 3.1 Exploratory Data Analysis (EDA) and data preprocessing

**Figure 1.**
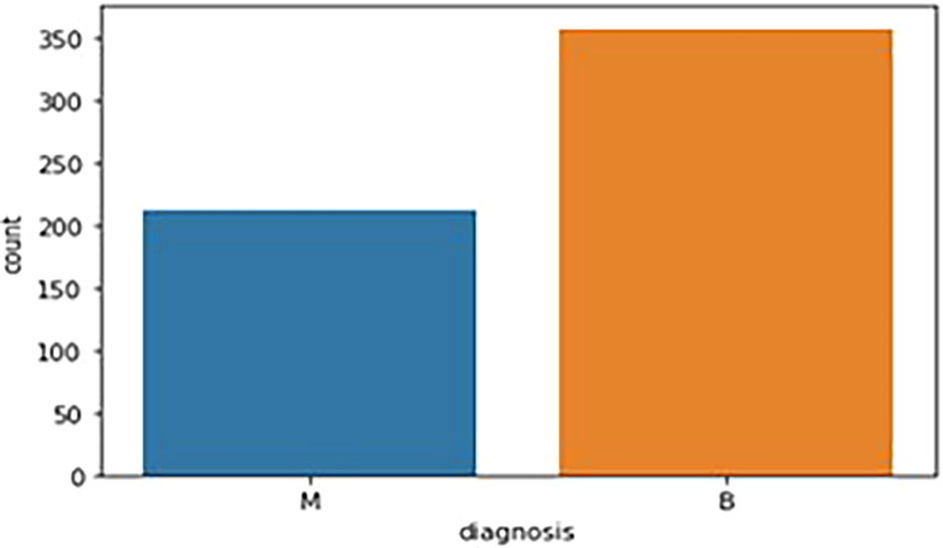
Bar graph showing the frequency of diagnosis column. M - Malignant Tumor and B - Benign Tumor.

The breast cancer dataset (569 samples, 32 features) underwent thorough exploratory data analysis (EDA) to assess structure and identify key features. Two redundant columns, “id” and “Unnamed: 32” (containing only NaN values), were removed during data cleaning. The target variable, “diagnosis,” was analyzed, revealing 59% malignant (M) and 41% benign (B) cases. A bar graph ( Figure 1) illustrates this distribution. Following data cleaning, the dataset was divided into feature variables (X) and the target variable (y), ensuring all numeric features remained in X while the categorical “diagnosis” variable was placed in y.

### 3.2 Feature extraction and visualization

**Figure 2.**
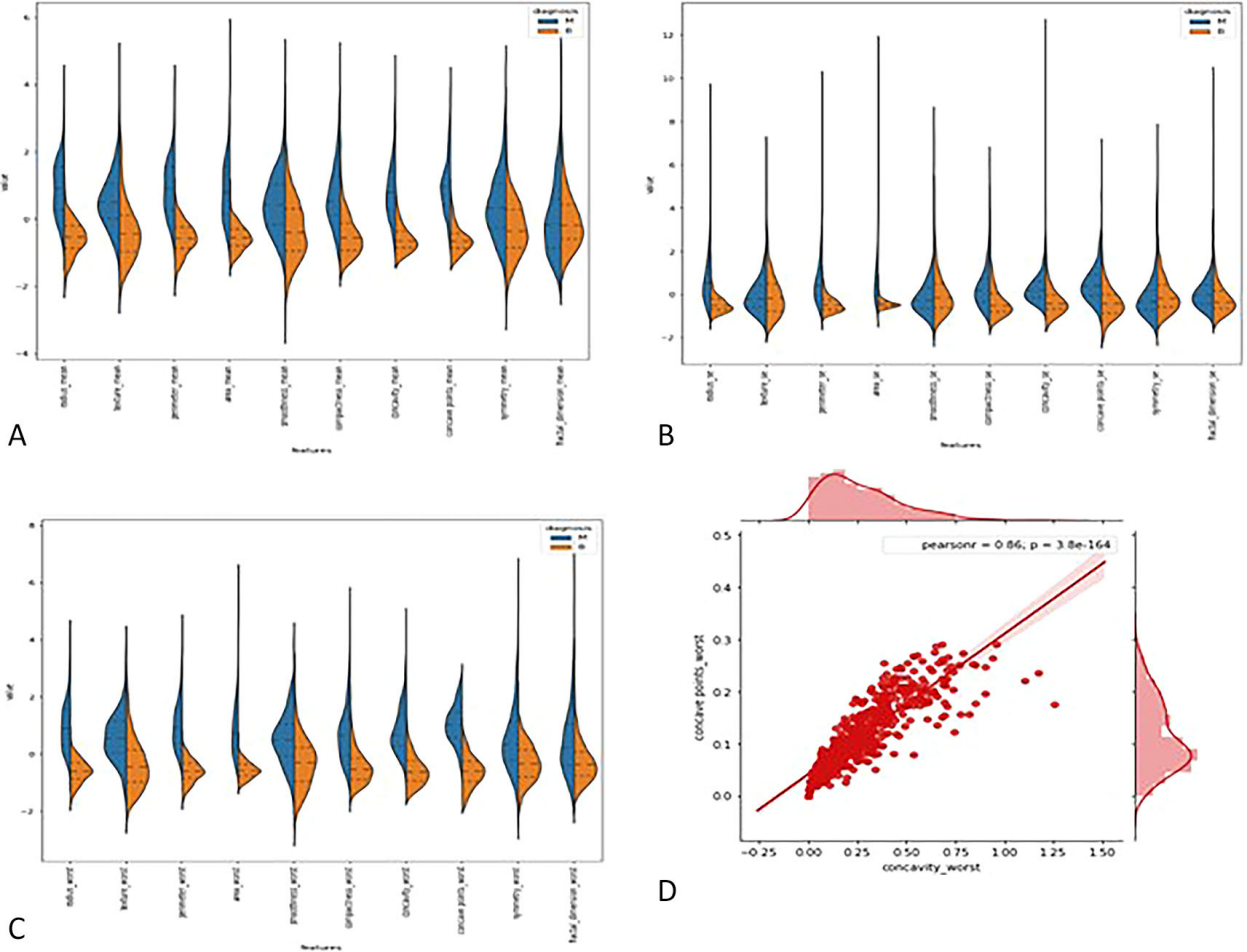
(A) Violin plot for first ten features (B) for second set of features (C) for last set of features (D) Joint plot for finding corelation between the concave wort and concavity worst.

**3.2.1 *Violin plots*: -** The distributions of the first thirty features in the dataset were visualized using violin plots to assess their potential for distinguishing between malignant and benign tumors. Key findings include the texture mean, which displayed distinct median values for the tumor types and a wider spread in the kernel density estimate (KDE) for malignant tumors, suggesting its potential as a useful feature for classification. In contrast, the fractal dimension mean showed similar medians for both tumor types, indicating limited discriminative power. Features such as concave points (se) and concavity (se) also exhibited overlapping distributions, making them less valuable for classification. On the other hand, area (se) demonstrated a clear separation between tumor types, highlighting its potential for classification. Similarly, the area (worst) feature showed a distinct separation between benign and malignant tumors, marking it as a strong candidate for classification models, whereas fractal dimension (worst) and concavity (worst) exhibited overlapping distributions, suggesting reduced utility. Overall, texture mean, area (se), and area (worst) emerged as the most promising features for classification, while the others showed limited differentiation between tumor types in the Figure 2 (A, B,C).

**3.2.2 *Joint plot*: -** A joint plot was used to analyze the relationship between concavity worst and concave points worst, as their distributions appeared to be similar. The joint plot, which combines scatter plots and histograms, provides a comprehensive view of the data’s distribution and the relationship between two variables. The analysis revealed a strong correlation of 0.86 between the two features, accompanied by a statistically significant p-value. This indicates a high degree of linear association between concavity worst and concave points worst, suggesting that they capture similar information regarding the tumor characteristics. Given their strong correlation, retaining only one of these features in the classification model is advisable, as including both would introduce redundancy and not contribute additional discriminative power in Figure 2(D).

**3.2.3 *Box plot***: - Box plots were used to visualize the distribution of key features across malignant and benign tumor groups, offering a clear representation of the data’s spread, central tendency, and variability. These plots divide the data into quartiles, highlighting the minimum, first quartile, median, third quartile, and maximum values, and can also identify potential outliers. Box plots are useful for comparing feature distributions between groups and identifying differences in spread and central values.

**Figure 3.**
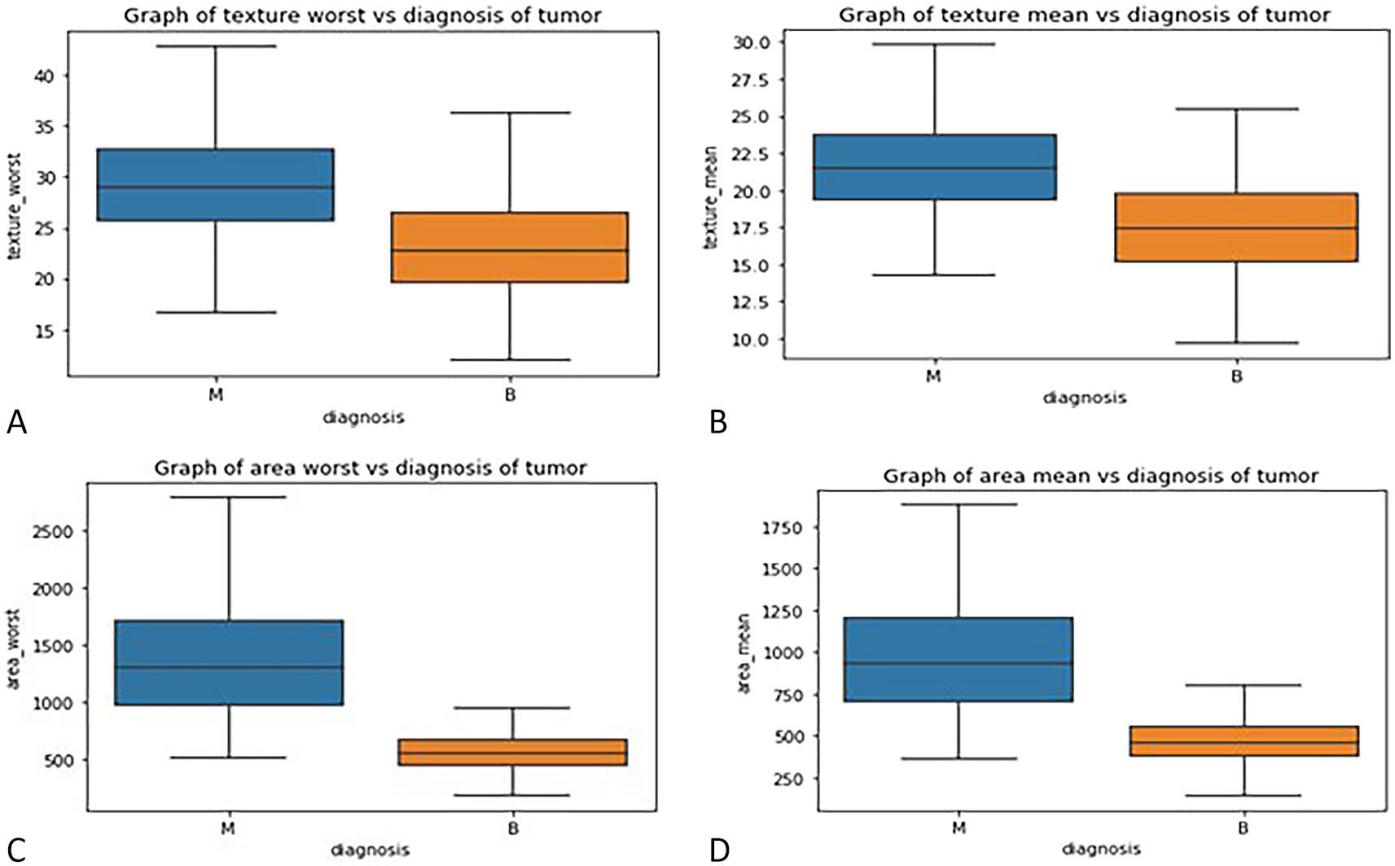
(A) Box plot graph of texture mean vs diagnosis of tumor (B) texture worse vs diagnosis of tumor (C) area mean vs diagnosis of tumor (D) area worst vs diagnosis of tumor.

In this study, box plots were employed to explore the relationship between highly correlated features in the correlation matrix, such as texture mean and texture worst, as well as area mean and area worst. The analysis of these features in relation to the diagnosis column revealed similar distributions for malignant and benign tumors, indicating redundancy in the information they provide. For instance, texture mean and texture worst showed comparable distributions, suggesting that retaining both features in the model would likely result in redundancy. Consequently, one of these highly correlated features can be excluded from the classification process without sacrificing predictive power. These insights were further validated through the visual examination of box plots, which helped clarify how each feature discriminates between malignant and benign groups in Figure 3(A, B, C, D).

### 3.3 Label encoding

Label encoding was employed to handle the categorical data within the dataset, specifically the diagnosis column, which consists of two classes: malignant (M) and benign (B). Label encoding is a technique used to transform categorical variables into numerical values, facilitating their inclusion in machine learning models that require numerical input. In this case, the diagnosis feature was encoded by assigning the value 0 to benign tumors and 1 to malignant tumors. This transformation of categorical data into binary values enables the classification algorithms to process the target variable effectively.

Label encoding is particularly useful for datasets with binary or ordinal categorical data, as it preserves the inherent order and structure of the classes. This method of encoding ensures that the diagnosis column can be used seamlessly in the machine learning models, enhancing the classification process and improving model performance. The encoded values (0 and 1) were then incorporated into the feature set, with the remaining extracted features, such as tumor radius, texture, perimeter, and others, remaining in their continuous form.

### 3.4 Dataset splitting and feature scaling

In this study, the dataset was divided into training and testing sets using the train-test split method to evaluate the performance of machine learning algorithms. The dataset was split with a 60:40 ratio, where 60% of the data was used for training the model, and 40% was reserved for testing. The primary goal of this split is to assess how well the model generalizes to unseen data by training it on the training set and evaluating it on the testing set. The training set allows the model to learn from known data, while the testing set is used exclusively for making predictions, providing an unbiased estimate of model performance.

The dataset was divided into input features (X) and the target variable (y). The target variable, diagnosis (benign or malignant), was assigned to y, and the remaining features used for classification were assigned to X. Consequently, the dataset was split into four variables: X train, X test, y train, and y test, representing the training and testing sets for both features and target variable.

Following the train-test split, feature scaling was performed to normalize the features within the dataset. Feature scaling is a preprocessing technique used to transform the features into a uniform scale, improving the performance of machine learning algorithms. In this study, the Robust Scaler was applied, which scales the data based on the interquartile range (IQR) while removing the median. This scaling method ensures that outliers have a minimal effect on the data, which is particularly beneficial when dealing with features that have different scales or units. The scaled data was then used for model training and evaluation, ensuring that all features contribute equally to the learning process.

### 3.5 Model development

In this study, various machine learning models were developed and evaluated using different supervised classification algorithms to identify the most accurate model for classifying benign and malignant breast lesions. A classifier algorithm is designed to map input data to specific categories, making it suitable for tasks such as classification of breast lesions. The algorithms utilized in this project include Logistic Regression, Support Vector Classifier (SVC) with a linear kernel, Support Vector Classifier (SVC) with a radial basis function (RBF) kernel, Decision Tree Classifier, and Random Forest Classifier.

The models were developed using the training dataset, with each classifier being imported from the learn library. The models were assigned to variables, and the fit method was used to train each model on the input features (X train) and target variable (y train). This method enabled the models to learn from the data and adjust their parameters accordingly to improve classification performance.

Following the training process, the accuracy of each model was calculated to assess their performance. The Decision Tree Classifier achieved the highest training accuracy of 1.0, indicating perfect classification performance on the training set. On the other hand, the SVC with the radial basis function kernel exhibited the lowest training accuracy among the classifiers. These results provide an indication of which models performed better in terms of training accuracy and highlight the potential for further model evaluation using additional metrics such as cross-validation, precision, recall, and F1 score to determine the most reliable classifier for the task.

### 3.6 Performance evaluation

The evaluation of the classification models was performed to determine their effectiveness in distinguishing between benign and malignant breast lesions. Testing accuracy was calculated using a confusion matrix, which summarizes the performance of the classification models in terms of actual and predicted values. The confusion matrix provided four key metrics: True Positive (TP), True Negative (TN), False Positive (FP), and False Negative (FN) values for each classification algorithm, as shown in Table 1.

**Table 1.** Confusion matrix values of the classification algorithms.

Classification Algorithm	True Positive (TP) Value	True Negative (TN) Value	False Positive (FP) Value	False Negative (FN) Value
Logistic Regression	142	83	2	2
SVC Linear	139	81	4	4
SVC RBF	142	83	1	2
Decision Tree	139	76	4	9
Random Forest	138	80	5	5

Among the classification algorithms, the Support Vector Classifier (SVC) with a Radial Basis Function (RBF) exhibited the highest testing accuracy of 0.986, indicating its superior ability to predict correctly. In contrast, the Decision Tree Classifier demonstrated the lowest testing accuracy of 0.942, suggesting room for improvement in its predictive capability.

### 3.7 Additional performance metrics

To further assess the quality of predictions, additional metrics such as precision, sensitivity (recall), F1 score, and specificity were calculated using the classification report function from the sklearn metrics package. These metrics evaluate the balance between true positive predictions and false positives/negatives, providing a comprehensive assessment of the classification algorithms The Support Vector Classifier with Radial Basis Function (SVC RBF) demonstrated the highest testing accuracy (0.9868) and consistently high precision, recall, F1 score, and specificity, establishing itself as the most robust classifier in this study. Logistic Regression performed comparably, achieving a testing accuracy of 0.9825, indicating reliable classification performance. In contrast, the Decision Tree Classifier, despite achieving the highest training accuracy (1.0), exhibited the lowest testing accuracy (0.9429), suggesting potential overfitting during training. The Random Forest classifier displayed a balanced performance, with a testing accuracy of 0.9561 and comparable metrics across precision, recall, and F1 score, making it a reliable but less optimal choice than SVC RBF and Logistic Regression in Table 2.

**Table 2.** Performance evaluation of the classification algorithm.

Classification Algorithms	Training Accuracy	Testing Accuracy	Final Accuracy	Precision		Sensitivity/Recall		F1 Score		Specificity/Support	
				“B”	“M”	“B”	“M”	“B”	“M”	“B”	“M”
Logistic Regression	0.9824	0.9825	0.98	0.99	0.98	0.99	0.98	0.99	0.98	143	85
SVC Linear	0.9883	0.9649	0.96	0.97	0.95	0.97	0.95	0.97	0.95	143	85
SVC RBF	0.9795	0.9868	0.99	0.99	0.99	0.99	0.98	0.99	0.98	143	85
Decision Tree	1.0	0.9429	0.94	0.94	0.95	0.97	0.89	0.96	0.92	143	85
Random Forest	0.9941	0.9561	0.96	0.97	0.94	0.97	0.94	0.97	0.94	143	85

SVC RBF - Support Vector Classifier with Radial Basis Function., B - Benign, M - Malignant.

## 4. Discussion

This study demonstrates the efficacy of machine learning techniques in the early detection and differential diagnosis of benign and malignant breast lesions, with the Support Vector Classifier using a Radial Basis Function (SVC-RBF) kernel emerging as the most accurate model. Achieving a remarkable accuracy of 99% on the Wisconsin Breast Cancer Diagnostic dataset, the SVC-RBF model exhibited superior precision (99% for benign and 98% for malignant cases), sensitivity (99% and 98% for benign and malignant cases, respectively), and specificity, with robust F1 scores for both classes. These results underscore its robustness and reliability in minimizing diagnostic errors, making it highly suited for clinical applications.

Exploratory data analysis (EDA), including violin plots, joint plots, and correlation matrices, revealed critical features such as texture mean, area (se), and area (worst), which were pivotal for classification. These insights enabled feature selection, improving the model’s accuracy while reducing redundancy. Comparatively, features like fractal dimension mean and concavity worst demonstrated limited diagnostic value.

The findings surpass prior studies in terms of model performance. For instance, M. Tahmooresi et al. ^18^ reported an SVM accuracy of 94%, while shen et al, ^19^ developed a deep learning algorithm for breast cancer detection on mammograms using an “end-to-end” approach, achieving high accuracy across heterogeneous datasets such as CBIS-DDSM (AUC: 0.91) and IN breast (AUC: 0.98). This improvement is attributed to advanced preprocessing techniques, such as robust scaling and hyperparameter tuning, combined with a comprehensive evaluation framework. Kayode et al. ^20^’s SVM model achieved a sensitivity of 94.4% and specificity of 91.3%, and Debelee et al. ^21^ reported 99% accuracy on the BGH dataset. While these results are comparable, this study’s comprehensive evaluation, including confusion matrix-derived metrics, adds rigor to the findings. Similarly, Suh et al. ^22^ explored neural network models, such as DenseNet-169 and EfficientNet-B5, achieving AUCs of 0.952–0.954. However, these models require larger datasets and computational resources, unlike the efficient SVC-RBF model used here. Notably, Viswanath et al. ^23^’s Random Forest model showed balanced performance (accuracy 84.84%, precision 90%, specificity 89%), yet it underperformed compared to the SVC-RBF model in this study, emphasizing the latter’s ability to capture non-linear relationships in high-dimensional datasets.

The SVC-RBF model offers significant advantages. Its transparency, facilitated by interpretability techniques and visual tools, ensures trust among clinicians, enhancing its potential as a decision-support tool. Moreover, the model’s efficiency in prioritizing high-risk cases and reducing diagnostic workloads aligns with the goal of improving patient outcomes. This study demonstrates the efficacy of machine learning techniques in the early detection and differential diagnosis of benign and malignant breast lesions, with the Support Vector Classifier using a Radial Basis Function (SVC-RBF) kernel emerging as the most accurate model.

However, it has some limitations, including the use of a limited and non-diverse dataset, which may affect the generalizability of the results. The complexity of the SVC-RBF model also poses challenges in terms of computational resources and interpretability. Future research should focus on expanding the dataset to include larger and more diverse populations, simplifying models to balance accuracy with interpretability, and exploring additional features and ensemble methods to enhance performance. Prospective clinical trials are necessary to validate the model’s effectiveness in real-world settings. Additionally, integrating mammography with other imaging modalities and developing user-friendly tools for clinicians can further improve diagnostic accuracy and support clinical decision-making.

## 5. Conclusion

Breast cancer diagnosis and treatment may be revolutionized by machine learning approaches that provide early detection, leading to more efficient therapeutic interventions. In a multi-centre study, larger datasets from different institutions can be accessed by applying different machine learning approaches. Early detection of breast cancer is key to slowing down the progression of the disease and reducing mortality rates. By leveraging the data from multiple institutions, machine learning can help identify breast cancer more quickly and accurately, leading to earlier intervention and better patient outcomes. With earlier intervention, the risk of mortality can be significantly reduced, leading to better patient outcomes and an overall improvement in public health.

## Ethics and consent

The dataset was extracted from the online open-source Wisconsin (Diagnostics) dataset. The study approval was obtained from Institutional Research Committee of Manipal College of Health Professions, Manipal on the 20 ^th^ of January 2022 (MCHP/Mpl/IRC/PG/2022/04). All procedures adhered to established ethical guidelines for secondary data analysis and data use policies. Consent is not applicable since the data was extracted from the online open source Wisconsin (Diagnostics) dataset.

## Data Availability

Kaggle: Wisconsin Breast Cancer Dataset, https://www.kaggle.com/datasets/uciml/breast-cancer-wisconsin-data The data sets of mammography with benign and malignant breast lesions. Data are available under the terms of the CC BY-NC-SA 4.0 (CC-BY 4.0).
