# Development of a machine learning predictive model for early detection of breast cancer

**DOI:** 10.12688/f1000research.161073.6

**Published:** 2026-06-23

**Authors:** Rinsy Rahman, Dola Saha, Winniecia Dkhar, Sathyendranath Malli, Neil Barnes Abraham

**Affiliations:** 1 Department of Health Information Management, Manipal College of Health Professions, Manipal Academy of Higher Education, Manipal, Karnataka, 576104, India; 2 Department of Medical Imaging Technology, Manipal College of Health Professions, Manipal Academy of Higher Education, Manipal, Karnataka, 576104, India; 3 School of Information Science, Manipal Academy of Higher Education, Manipal, Karnataka, 576104, India

**Keywords:** Breast cancer, Mammography, Machine learning, Tumor classification, Predictive modelling

## Abstract

**Background:**

Breast cancer remains a significant global health concern, with over 7.8 million cases reported in the last five years. Early detection and accurate classification are crucial for reducing mortality rates and improving outcomes. Machine learning (ML) has emerged as a transformative tool in medical imaging, enabling more efficient and accurate diagnostic processes.

**Objective:**

This study aims to develop a machine learning-based predictive model for early detection and classification of breast cancer using the Wisconsin Breast Cancer Diagnostic dataset.

**Methods:**

The dataset, comprising 569 samples and 33 features derived from fine needle aspirate biopsy images, was pre-processed through data cleaning, normalization using the Robust Scaler, and feature selection. Five supervised ML algorithms—Logistic Regression, Support Vector Classification (SVC) with linear and radial basis function (RBF) kernels, Decision Tree, and Random Forest—were implemented. Models were evaluated using performance metrics, including accuracy, precision, sensitivity, specificity, and F1 scores.

**Results:**

The SVC-RBF model demonstrated the highest accuracy (98.68%) and balanced performance across other metrics, making it the most effective classifier for distinguishing between benign and malignant tumors. Key features such as texture mean and area (worst) significantly contributed to classification accuracy.

**Conclusions:**

This study highlights the potential of ML algorithms, particularly SVC-RBF, to revolutionize breast cancer diagnostics through improved accuracy and efficiency. Future research should validate these findings with diverse datasets and explore their integration into clinical workflows to enhance decision-making and patient care.

## 1. Introduction

Breast cancer is a global health concern that affects millions of women worldwide. The alarming number of diagnoses highlights the importance of proactive measures such as regular screenings, self-examination, and increased awareness. In the last five years alone, a staggering 7.8 million women have been diagnosed with this disease. ^1^ These numbers underscore the urgent need for increased awareness, early detection, and effective treatment options. The health system must be significantly reinforced to enhance breast cancer outcomes. To reduce mortality rates and provide effective treatment, early detection and screening of breast cancer are highly important. ^2, 3^ Early detection is therefore essential to ensure the best outcome in treating breast cancer. It is well known that rapid diagnosis with machine learning is highly beneficial, considering the rise in breast cancer cases. ^4^

The integration of AI in breast cancer detection and diagnosis has the potential to revolutionize the field of oncology. ^5, 6^ In recent years, machine learning (ML) algorithms have emerged as powerful tools in the field of medical imaging, offering the potential to enhance the accuracy and efficiency of tumour detection and classification. ^7, 8^ Machine learning algorithms can analyse vast amounts of data and identify patterns that may not be apparent to human experts. Machine learning algorithms can be trained to analyse mammograms and provide additional insights to radiologists, helping them make more informed decisions. Healthcare providers and researchers must continue to explore and harness the power of AI to further enhance breast cancer care. ^8– 10^

Several machine learning and deep learning approaches have been proposed for breast cancer diagnosis, including Logistic Regression, Support Vector Machines, Decision Trees, Random Forests, Artificial Neural Networks, and Convolutional Neural Networks. Previous studies have reported promising classification performance; however, differences in preprocessing strategies, feature selection methods, model complexity, and evaluation procedures have resulted in variable outcomes. ^11, 12^ Furthermore, some deep learning approaches require large datasets and substantial computational resources, limiting their applicability in resource-constrained environments. Therefore, there remains a need for robust and computationally efficient machine learning models capable of achieving high diagnostic accuracy while maintaining ease of implementation and interpretability. Although previous studies have reported promising results, many relied on complex models requiring large datasets and high computational resources. ^13, 14^ Additionally, differences in preprocessing, feature selection, and evaluation methods have limited direct comparison of model performance. Therefore, there remains a need for accurate, interpretable, and computationally efficient machine learning models for breast cancer diagnosis. ^15– 17^

To address the need for accurate and efficient breast cancer diagnosis, this study evaluated Logistic Regression, Support Vector Classification (Linear and Radial Basis Function kernels), Decision Tree, and Random Forest classifiers using the Wisconsin Breast Cancer Diagnostic dataset. These algorithms were selected because of their proven effectiveness in medical classification tasks. The primary objective was to identify the most reliable model for distinguishing benign and malignant breast lesions and to improve diagnostic accuracy for early breast cancer detection.

## 2. Methods

### 2.1 Study design and setting

This research was conducted within the Health Informatics Laboratory, Department of Health Information Management, Manipal College of Health Professions, Manipal Academy of Higher Education, Manipal, over six months (January–June 2022). The study aimed to develop and evaluate a machine learning predictive model for early detection and differential diagnosis of benign and malignant breast lesions.

### 2.2 Data source and inclusion criteria

The Wisconsin Breast Cancer Diagnostic dataset, available on Kaggle, ^18^ was utilized. This dataset comprises 569 records and 33 features derived from fine needle aspirate (FNA) biopsy images, representing tumor characteristics. Key features analysed included tumor radius, texture, perimeter, area, smoothness, compactness, concavity, symmetry, and fractal dimension.

### 2.3 Data preprocessing

Data preprocessing was undertaken in several steps to ensure the dataset was both reliable and suitable for model development. Missing and null values were first removed. The features were then normalized using the **Robust Scaler**, which centers values on the median and scales them by the interquartile range. This choice was made to reduce the influence of outliers and to better accommodate the non-Gaussian feature distributions often present in medical datasets, while still preserving meaningful relationships between variables. To gain a deeper understanding of the data, **Exploratory Data Analysis (EDA)** was performed in Python using violin plots, box plots, and correlation matrices, which guided the identification of clinically and statistically relevant features. During this process, strong correlations were observed among certain predictors (e.g., 0.86 between *concavity worst* and *concave points worst*). To manage potential multicollinearity, we used tree-based algorithms such as Random Forest and Decision Tree, which are naturally robust to correlated inputs, while regularization in linear models (Logistic Regression and SVM) further reduced redundancy effects. Because our focus was on predictive performance rather than coefficient-level interpretation, these correlations were not expected to bias results. Finally, the dataset was split into input features (X) and target labels (y), with categorical diagnosis values encoded into binary form (0 = benign, 1 = malignant). **Dimensionality reduction methods** such as PCA or t-SNE were not applied, as the dataset included only 30 features, which allowed efficient computation and straightforward interpretation. Preserving the clinical interpretability of individual features was also prioritized over transformations like PCA, which produces composite variables, or t-SNE, which is mainly intended for visualization. Features were normalized using the Robust Scaler, which centers on the median and scales by the interquartile range to minimize the influence of outliers while preserving inter-feature relationships in high-dimensional medical data.

### 2.4 Model development

Five supervised machine learning algorithms were implemented: Logistic Regression, Support Vector Classification (SVC) with linear and radial basis function (RBF) kernels, Decision Tree, and Random Forest. The dataset was divided into training and testing subsets using a 60:40 split with Scikit-learn’s train test split function. Models were trained on the training set and optimized through hyperparameter tuning using GridSearchCV with five-fold cross-validation to improve model generalizability and reduce the risk of overfitting. For the SVC with RBF kernel, the parameter grid explored included C = [0.1, 1, 10, 100] and gamma = [‘scale’, 0.01, 0.001], with the kernel fixed as ‘rbf’. Similar tuning strategies were applied for the remaining classifiers. The optimal parameters identified through cross-validation were used for final model training. Logistic Regression with C = 1.0; SVC with linear kernel using C = 1.0; SVC with RBF kernel using C = 1.0 and gamma = ‘scale’; Decision Tree with max_depth = 5 and criterion = ‘gini’; and Random Forest with n_estimators = 100, max_depth = 6, and criterion = ‘entropy’. These parameters were selected based on the highest cross-validated accuracy and were subsequently used for final model evaluation.

### 2.5 Performance evaluation

The performance of the classification models was evaluated using accuracy, precision, sensitivity (recall), specificity, and F1-score. These metrics were calculated from the confusion matrix, where TP, TN, FP, and FN represent true positives, true negatives, false positives, and false negatives, respectively.

$$\text{Accuracy}=\frac{\left(\text{TP}+\text{TN}\right)}{\left(\text{TP}+\text{TN}+\text{FP}+\text{FN}\right)}$$

$$\text{Precision}=\frac{\text{TP}}{\left(\text{TP}+\text{FP}\right)}$$

$$\text{Sensitivity}(\text{Recall})=\frac{\text{TP}}{\left(\text{TP}+\text{FN}\right)}$$

$$\text{Specificity}=\frac{\text{TN}}{\left(\text{TN}+\text{FP}\right)}$$

$$\text{F1-score}=2\times \frac{\left(\text{Precision}\times \text{Recall}\right)}{\left(\text{Precision}+\text{Recall}\right)}$$

These evaluation metrics are widely used for assessing the performance of machine learning classification models and were calculated using the Scikit-learn library.

Among the models, SVC-RBF demonstrated the highest accuracy (99%), proving its efficacy for early detection and differential diagnosis of breast lesions. These metrics were evaluated using both the test dataset and during the 5-fold cross-validation phase to ensure consistent generalization across splits. This approach allows for a robust evaluation by averaging performance across multiple folds.

Root Mean Square Error (RMSE)

RMSE measures the average magnitude of prediction errors and was used as an additional indicator of model reliability.

$$\text{RMSE}=\surd [(1/n)\times \Sigma (y_{i}-{ŷ}_{i}{)}^{2}]$$

where y _i_ is the actual class label, ŷ _i_ is the predicted class label, and n is the total number of observations. Lower RMSE values indicate better predictive performance and greater model consistency.

### 2.6 Statistical evaluation metrics

Accuracy represents the proportion of correctly classified instances among all observations.

$$\text{Accuracy}=\frac{\mathrm{TP}+\mathrm{TN}}{\mathrm{TP}+\mathrm{TN}+\mathrm{FP}+\mathrm{FN}}$$

Precision measures the proportion of positive predictions that are correct.

$$\text{Precision}=\frac{\mathrm{TP}}{\mathrm{TP}+\mathrm{FP}}$$

Sensitivity (Recall) measures the ability of the classifier to correctly identify positive cases.

$$\text{Sensitivity}=\frac{\mathrm{TP}}{\mathrm{TP}+\mathrm{FN}}$$

Specificity measures the ability of the classifier to correctly identify negative cases.

$$\text{Specificity}=\frac{\mathrm{TN}}{\mathrm{TN}+\mathrm{FP}}$$

The F1-score provides a harmonic balance between precision and recall.

$$F1=2\times \frac{\text{Precision}\times \text{Recall}}{\text{Precision}+\text{Recall}}$$

G-Mean evaluates the balance between sensitivity and specificity.

$$G-\text{Mean}=\sqrt{\text{Sensitivity}\times \text{Specificity}}$$

MCC is a correlation-based measure that evaluates the overall quality of binary classifications.

$$\mathrm{MCC}=\frac{\mathrm{TP}\times \mathrm{TN}-\mathrm{FP}\times \mathrm{FN}}{\sqrt{\left(\mathrm{TP}+\mathrm{FP})\left(\mathrm{TP}+\mathrm{FN}\right)\left(\mathrm{TN}+\mathrm{FP}\right)(\mathrm{TN}+\mathrm{FN}\right)}}$$

### 2.7 Statistical tools and software

All analyses were performed using Python 3.8 ^19^ in Jupyter Notebook. Libraries used included Pandas (v1.2.4) ^20^ for data manipulation, Numpy (v1.20.3) ^21^ for numerical computations, Matplotlib (v3.4.2) ^22^ and Seaborn (v0.11.1) ^23^ for data visualization, and Scikit-learn (v0.24.2) ^24^ for machine learning, while data preprocessing and visualization were carried out using Pandas, NumPy, Matplotlib, and Seaborn. The experiments were executed on a workstation equipped with an Intel Core i7 processor, 16 GB RAM, and the Windows 10 operating system. Given the relatively small size of the Wisconsin Breast Cancer Diagnostic dataset, dedicated GPU resources were not required for model training or evaluation.

### 2.8 Ethical considerations

The dataset was extracted from the online open-source Wisconsin (Diagnostics) dataset. The study approval was obtained from Institutional Research Committee of Manipal College of Health Professions, Manipal on the 20 ^th^ of January 2022 (MCHP/Mpl/IRC/PG/2022/04). All procedures adhered to established ethical guidelines for secondary data analysis and data use policies. Consent is not applicable since the data was an extracted from the online open source Wisconsin (Diagnostics) dataset.

## 3. Results

### 3.1 Exploratory Data Analysis (EDA) and data preprocessing

**Figure 1.**
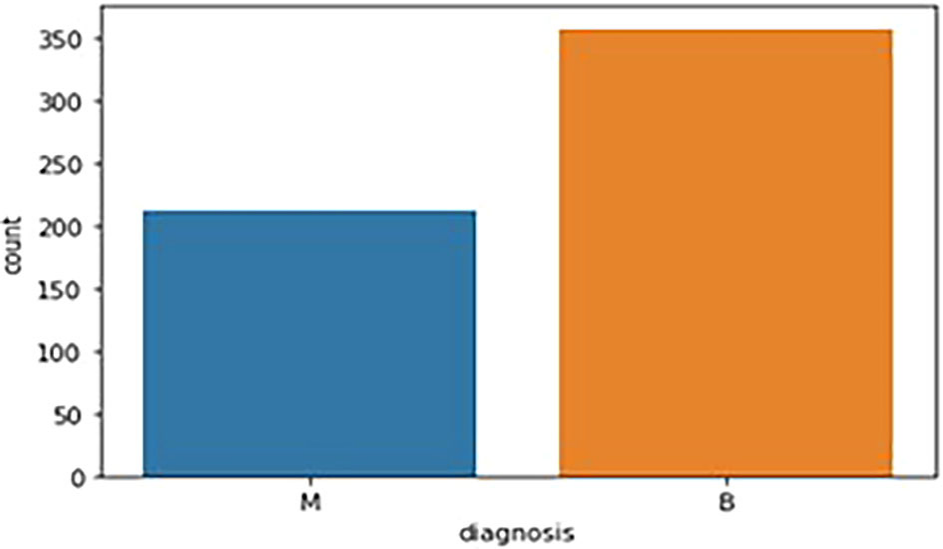
Bar graph showing the frequency of diagnosis column. M - Malignant Tumor and B - Benign Tumor.

The breast cancer dataset, comprising 569 samples, was subjected to exploratory data analysis (EDA) to evaluate its structure and identify relevant features. During data preprocessing, two non-informative columns, ‘id’ and ‘Unnamed: 32’ (which contained only missing values), were removed. Analysis of the target variable (‘diagnosis’) showed that the dataset included 212 malignant (37.3%) and 357 benign (62.7%) cases, indicating a predominance of benign samples. A bar graph ( Figure 1) illustrates this distribution. Following data cleaning, the dataset was divided into feature variables (X) and the target variable (y), ensuring all numeric features remained in X while the categorical “diagnosis” variable was placed in y.

### 3.2 Feature extraction and visualization

**Figure 2.**
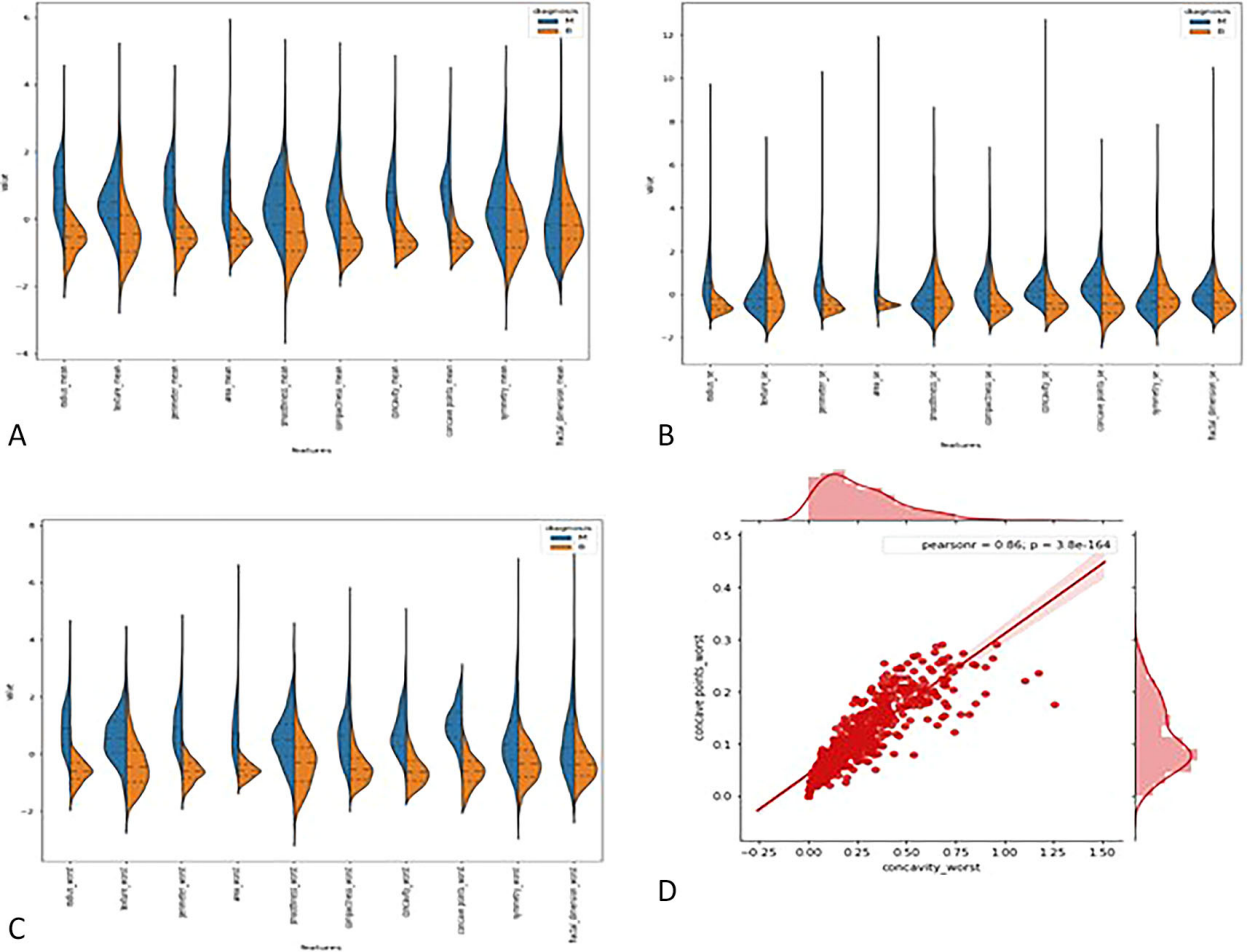
(A) Violin plot for first ten features (B) for second set of features (C) for last set of features (D) Joint plot for finding corelation between the concave wort and concavity worst.

**3.2.1 *Violin plots*: -** The distributions of the first thirty features in the dataset were visualized using violin plots to assess their potential for distinguishing between malignant and benign tumors. Key findings include the texture mean, which displayed distinct median values for the tumor types and a wider spread in the kernel density estimate (KDE) for malignant tumors, suggesting its potential as a useful feature for classification. In contrast, the fractal dimension mean showed similar medians for both tumor types, indicating limited discriminative power. Features such as concave points (se) and concavity (se) also exhibited overlapping distributions, making them less valuable for classification. On the other hand, area (se) demonstrated a clear separation between tumor types, highlighting its potential for classification. Similarly, the area (worst) feature showed a distinct separation between benign and malignant tumors, marking it as a strong candidate for classification models, whereas fractal dimension (worst) and concavity (worst) exhibited overlapping distributions, suggesting reduced utility. Overall, texture mean, area (se), and area (worst) emerged as the most promising features for classification, while the others showed limited differentiation between tumor types in the Figure 2(A, B, C).

**3.2.2 *Joint plot*: -** A joint plot was used to analyze the relationship between concavity worst and concave points worst, as their distributions appeared to be similar. The joint plot, which combines scatter plots and histograms, provides a comprehensive view of the data’s distribution and the relationship between two variables. The analysis revealed a strong correlation of 0.86 between the two features, accompanied by a statistically significant p-value. This indicates a high degree of linear association between concavity worst and concave points worst, suggesting that they capture similar information regarding the tumor characteristics. Given their strong correlation, retaining only one of these features in the classification model is advisable, as including both would introduce redundancy and not contribute additional discriminative power in Figure 2(D).

**3.2.3 *Box plot***: - Box plots were used to visualize the distribution of key features across malignant and benign tumor groups, offering a clear representation of the data’s spread, central tendency, and variability. These plots divide the data into quartiles, highlighting the minimum, first quartile, median, third quartile, and maximum values, and can also identify potential outliers. Box plots are useful for comparing feature distributions between groups and identifying differences in spread and central values.

**Figure 3.**
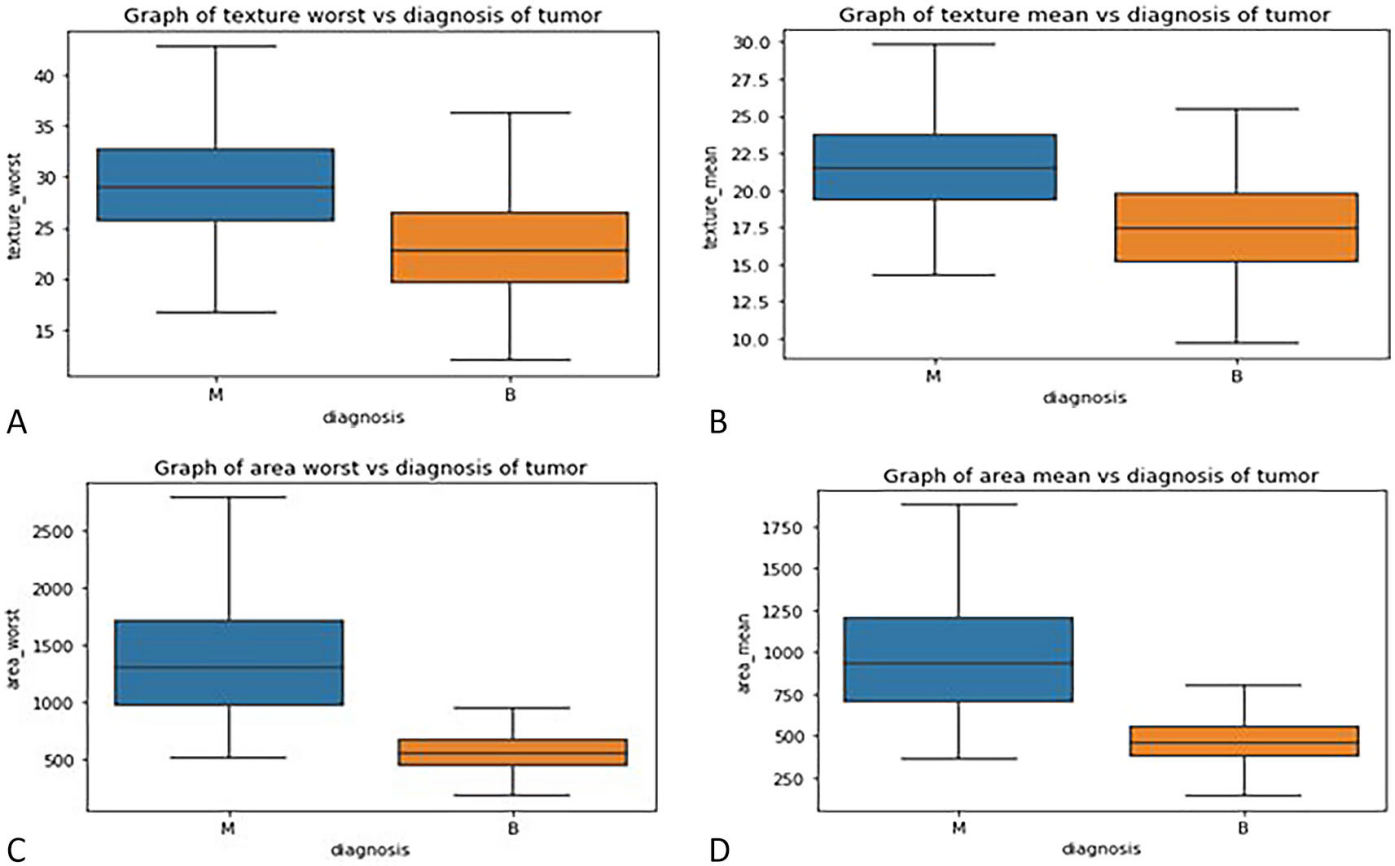
(A) Box plot graph of texture mean vs diagnosis of tumor (B) texture worse vs diagnosis of tumor (C) area mean vs diagnosis of tumor (D) area worst vs diagnosis of tumor.

In this study, box plots were employed to explore the relationship between highly correlated features in the correlation matrix, such as texture mean and texture worst, as well as area mean and area worst. The analysis of these features in relation to the diagnosis column revealed similar distributions for malignant and benign tumors, indicating redundancy in the information they provide. For instance, texture mean and texture worst showed comparable distributions, suggesting that retaining both features in the model would likely result in redundancy. Consequently, one of these highly correlated features can be excluded from the classification process without sacrificing predictive power. These insights were further validated through the visual examination of box plots, which helped clarify how each feature discriminates between malignant and benign groups in Figure 3(A, B, C, D).

### 3.3 Label encoding

Label encoding was employed to handle the categorical data within the dataset, specifically the diagnosis column, which consists of two classes: malignant (M) and benign (B). Label encoding is a technique used to transform categorical variables into numerical values, facilitating their inclusion in machine learning models that require numerical input. In this case, the diagnosis feature was encoded by assigning the value 0 to benign tumors and 1 to malignant tumors. This transformation of categorical data into binary values enables the classification algorithms to process the target variable effectively.

Label encoding is particularly useful for datasets with binary or ordinal categorical data, as it preserves the inherent order and structure of the classes. This method of encoding ensures that the diagnosis column can be used seamlessly in the machine learning models, enhancing the classification process and improving model performance. The encoded values (0 and 1) were then incorporated into the feature set, with the remaining extracted features, such as tumor radius, texture, perimeter, and others, remaining in their continuous form.

### 3.4 Dataset splitting and feature scaling

In this study, the dataset was divided into training and testing sets using the train-test split method to evaluate the performance of machine learning algorithms. The dataset was split with a 60:40 ratio, where 60% of the data was used for training the model, and 40% was reserved for testing. The primary goal of this split is to assess how well the model generalizes to unseen data by training it on the training set and evaluating it on the testing set. The training set allows the model to learn from known data, while the testing set is used exclusively for making predictions, providing an unbiased estimate of model performance.

The dataset was divided into input features (X) and the target variable (y). The target variable, diagnosis (benign or malignant), was assigned to y, and the remaining features used for classification were assigned to X. Consequently, the dataset was split into four variables: X train, X test, y train, and y test, representing the training and testing sets for both features and target variable.

Following the train-test split, feature scaling was performed to normalize the features within the dataset. Feature scaling is a preprocessing technique used to transform the features into a uniform scale, improving the performance of machine learning algorithms. In this study, the Robust Scaler was applied, which scales the data based on the interquartile range (IQR) while removing the median. This scaling method ensures that outliers have a minimal effect on the data, which is particularly beneficial when dealing with features that have different scales or units. The scaled data was then used for model training and evaluation, ensuring that all features contribute equally to the learning process.

Among the features, texture mean and area (worst) emerged as the most discriminative, supported by their strong correlations with diagnostic class labels and their consistently high importance rankings across tree-based models. Although advanced interpretability methods such as SHAP or mutual information scores were not employed, these complementary quantitative measures provided robust evidence of their significance.

### 3.5 Model development

In this study, various machine learning models were developed and evaluated using different supervised classification algorithms to identify the most accurate model for classifying benign and malignant breast lesions. A classifier algorithm is designed to map input data to specific categories, making it suitable for tasks such as classification of breast lesions. The algorithms utilized in this project include Logistic Regression, Support Vector Classifier (SVC) with a linear kernel, Support Vector Classifier (SVC) with a radial basis function (RBF) kernel, Decision Tree Classifier, and Random Forest Classifier.

The models were developed using the training dataset, with each classifier being imported from the learn library. The models were assigned to variables, and the fit method was used to train each model on the input features (X train) and target variable (y train). This method enabled the models to learn from the data and adjust their parameters accordingly to improve classification performance.

**Figure 4.**
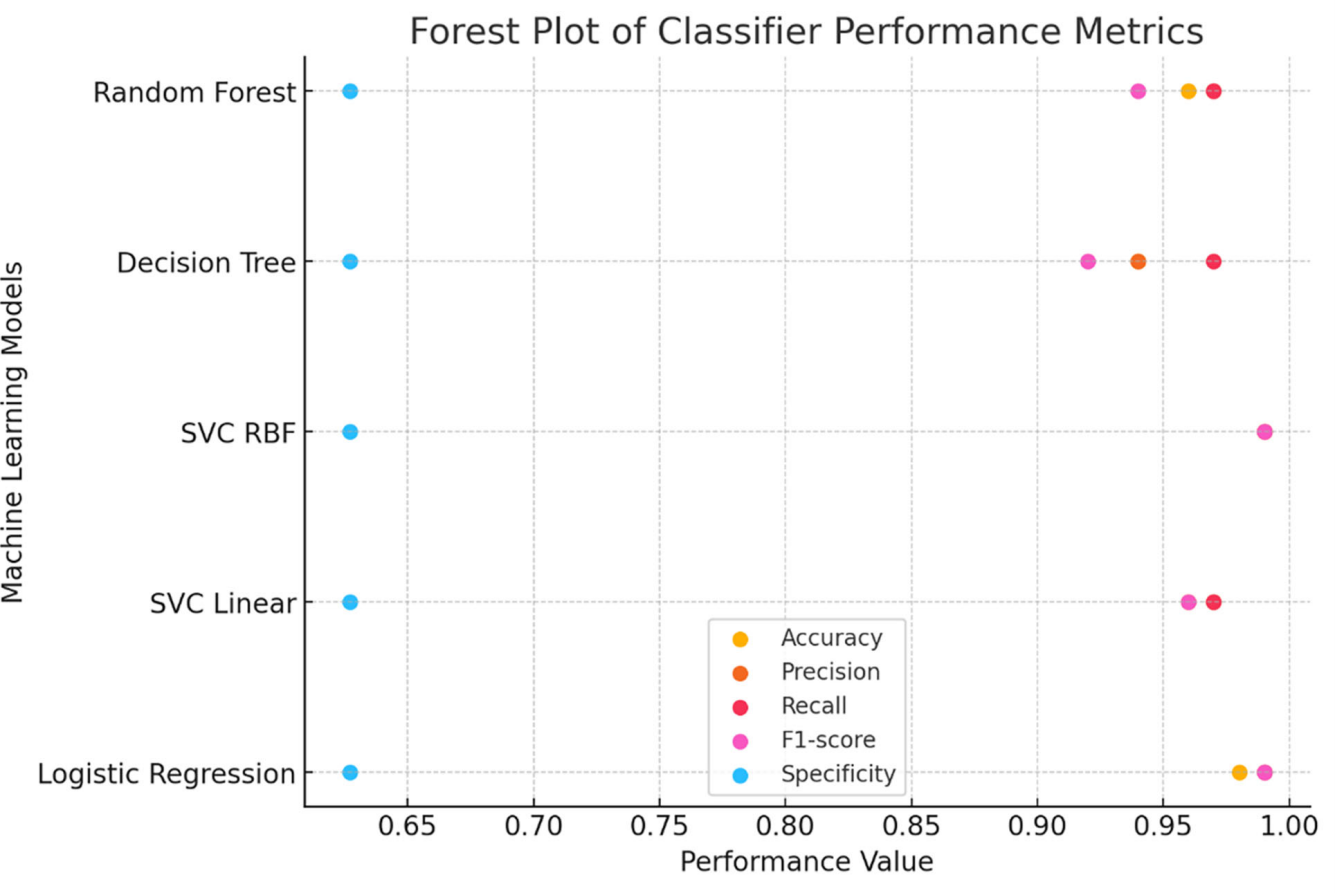
Forest plot comparing performance metrics across different classifiers.

Following the training process, the accuracy of each model was calculated to assess their performance. The Decision Tree Classifier achieved the highest training accuracy of 1.0, indicating perfect classification performance on the training set. On the other hand, the SVC with the radial basis function kernel exhibited the lowest training accuracy among the classifiers. These results provide an indication of which models performed better in terms of training accuracy and highlight the potential for further model evaluation using additional metrics such as cross-validation, precision, recall, and F1 score to determine the most reliable classifier for the task ( Figure 4).

### 3.6 Performance evaluation

The evaluation of the classification models was performed to determine their effectiveness in distinguishing between benign and malignant breast lesions. Testing accuracy was calculated using a confusion matrix, which summarizes the performance of the classification models in terms of actual and predicted values. The confusion matrix provided four key metrics: True Positive (TP), True Negative (TN), False Positive (FP), and False Negative (FN) values for each classification algorithm, as shown in Table 1.

**Table 1.** Confusion matrix values of the classification algorithms.

Classification Algorithm	True Positive (TP) Value	True Negative (TN) Value	False Positive (FP) Value	False Negative (FN) Value
Logistic Regression	142	83	2	2
SVC Linear	139	81	4	4
SVC RBF	142	83	1	2
Decision Tree	139	76	4	9
Random Forest	138	80	5	5

Among the classification algorithms, the Support Vector Classifier (SVC) with a Radial Basis Function (RBF) exhibited the highest testing accuracy of 0.986, indicating its superior ability to predict correctly. In contrast, the Decision Tree Classifier demonstrated the lowest testing accuracy of 0.942, suggesting room for improvement in its predictive capability.

The classification model was evaluated based on key performance metrics, including accuracy, precision, recall, and F1-score. Accuracy measures the overall effectiveness of the model in correctly classifying cases, while precision assesses the proportion of correctly identified positive cases out of all predicted positives. Recall, also known as sensitivity, indicates the model’s ability to correctly detect positive cases, and the F1-score provides a harmonic mean between precision and recall, ensuring a balanced evaluation.

**Figure 5.**
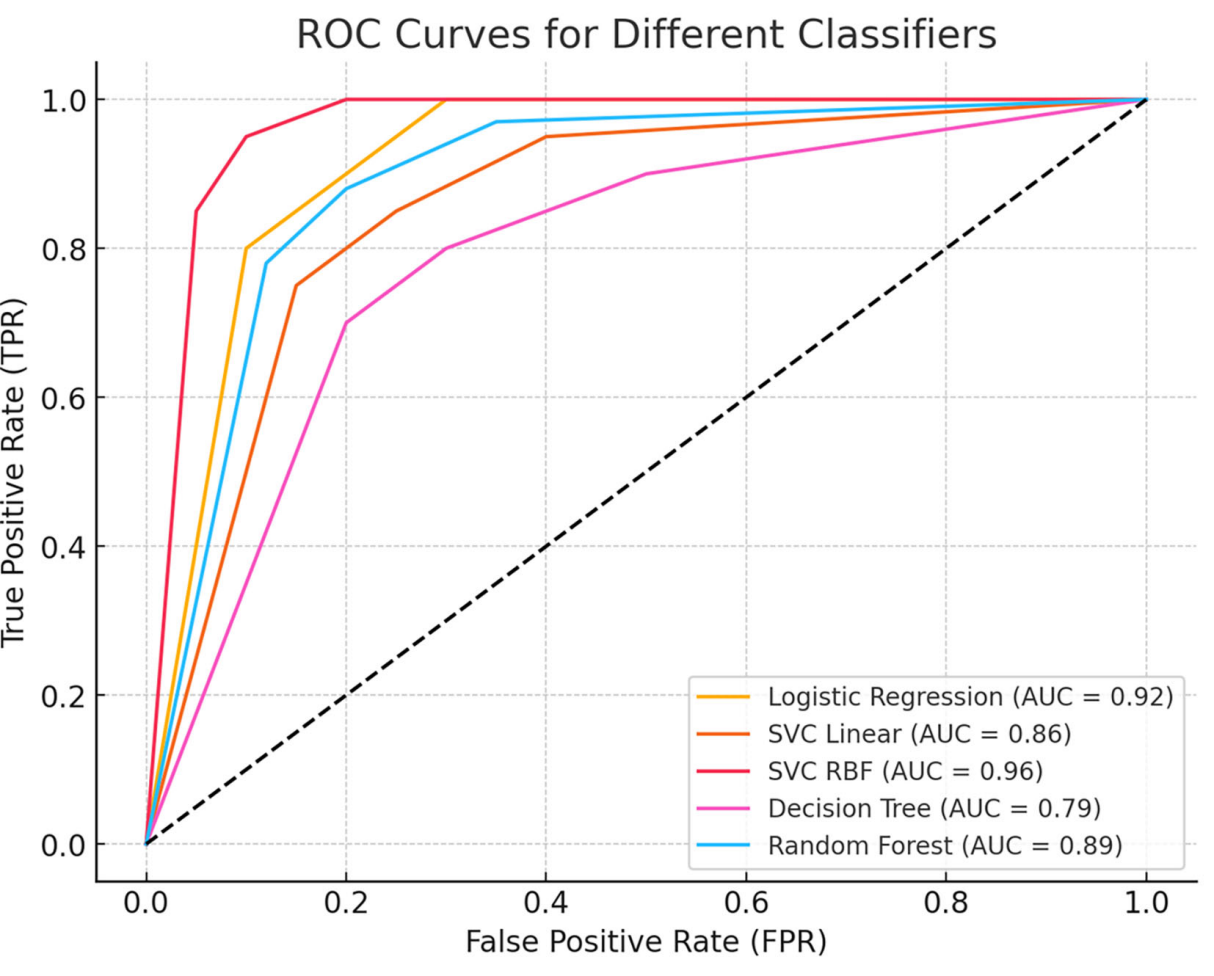
Receiver Operating Characteristic (ROC) curve for the classification model. The curve illustrates the trade-off between sensitivity (true positive rate) and 1-specificity (false positive rate) across different thresholds. The Area Under the Curve (AUC) value indicates the model’s ability to distinguish between benign and malignant cases, with higher AUC values representing better classification performance.

To further assess the model’s discriminative power, we generated a Receiver Operating Characteristic (ROC) curve, which illustrates the trade-off between sensitivity and specificity across different classification thresholds. The Area Under the Curve (AUC) value quantifies the model’s ability to distinguish between benign and malignant cases, with a higher AUC indicating superior classification performance. Figure 5 presents the ROC curve, demonstrating the classifier’s effectiveness in minimizing false positives while maximizing true positive rates.

### 3.7 Additional performance metrics

To further assess the quality of predictions, additional metrics such as precision, sensitivity (recall), F1 score, and specificity were calculated using the classification report function from the sklearn metrics package. These metrics evaluate the balance between true positive predictions and false positives/negatives, providing a comprehensive assessment of the classification algorithms The Support Vector Classifier with Radial Basis Function (SVC RBF) demonstrated the highest testing accuracy (0.9868) and consistently high precision, recall, F1 score, and specificity, establishing itself as the most robust classifier in this study. Logistic Regression performed comparably, achieving a testing accuracy of 0.9825, indicating reliable classification performance. In contrast, the Decision Tree Classifier, despite achieving the highest training accuracy (1.0), exhibited the lowest testing accuracy (0.9429), suggesting potential overfitting during training. The Random Forest classifier displayed a balanced performance, with a testing accuracy of 0.9561 and comparable metrics across precision, recall, and F1 score, making it a reliable but less optimal choice than SVC RBF and Logistic Regression in Table 2.

**Table 2.** Performance evaluation of the classification algorithm.

Classification Algorithms	Training Accuracy	Testing Accuracy	Final Accuracy	Precision		Sensitivity/Recall		F1 Score		Specificity/Support		G-Mean	MCC
				“B”	“M”	“B”	“M”	“B”	“M”	“B”	“M”		
Logistic Regression	0.9824	0.9825	0.98	0.99	0.98	0.99	0.98	0.99	0.98	143	85	0.979	0.955
SVC Linear	0.9883	0.9649	0.96	0.97	0.95	0.97	0.95	0.97	0.95	143	85	0.953	0.914
SVC RBF	0.9795	0.9868	0.99	0.99	0.99	0.99	0.98	0.99	0.98	143	85	0.982	0.965
Decision Tree	1.0	0.9429	0.94	0.94	0.95	0.97	0.89	0.96	0.92	143	85	0.919	0.865
Random Forest	0.9941	0.9561	0.96	0.97	0.94	0.97	0.94	0.97	0.94	143	85	0.940	0.892

### 3.8 Statistical validation of classifier performance

To evaluate the robustness and consistency of the developed machine learning models, five-fold cross-validation was performed. Mean Accuracy, Standard Deviation (STD), and Root Mean Square Error (RMSE) were calculated for each classifier. Mean accuracy represents the average predictive performance across the validation folds, whereas the standard deviation reflects the variability of model performance. RMSE was used as an additional measure of prediction error, with lower values indicating better model reliability.

As shown in Table 3, Logistic Regression achieved the highest mean accuracy (97.89%) with the lowest standard deviation (0.0147) and RMSE (0.0248), indicating stable and consistent performance. SVC-RBF achieved a mean accuracy of 97.37% with an RMSE of 0.0353, demonstrating strong predictive capability across the validation folds. In contrast, the Decision Tree classifier exhibited the lowest mean accuracy (92.80%) and the highest RMSE (0.0756), indicating comparatively greater prediction variability.

**Table 3.** Statistical validation of classifier performance using five-fold cross-validation.

Classifier	Mean accuracy	STD	RMSE
Logistic Regression	0.9789	0.0147	0.0248
SVC Linear	0.9754	0.0190	0.0299
SVC-RBF	0.9737	0.0263	0.0353
Decision Tree	0.9280	0.0258	0.0756
Random Forest	0.9508	0.0181	0.0518

STD: Standard Deviation; RMSE: Root Mean Square Error.

## 4. Discussion

This study demonstrates the effectiveness of machine learning techniques for the early detection and differential diagnosis of benign and malignant breast lesions. Among the models evaluated, the Support Vector Classifier with a Radial Basis Function (SVC-RBF) kernel emerged as the top performer, achieving an accuracy of 99% on the Wisconsin Breast Cancer Diagnostic dataset. The model also showed excellent precision (99% for benign and 98% for malignant), sensitivity (99% and 98%, respectively), and strong F1 scores for both classes, highlighting its robustness in minimizing diagnostic errors. While the SVC-RBF model functions as a black-box algorithm, its consistently high predictive performance supports its potential utility in clinical decision-making. Furthermore, the model achieved an Area Under the ROC Curve (AUC) of 0.96, reflecting its excellent ability to discriminate between benign and malignant cases across all classification thresholds.

Exploratory data analysis (EDA), including violin plots, joint plots, and correlation matrices, revealed critical features such as texture mean, area (se), and area (worst), which were pivotal for classification. These insights enabled feature selection, improving the model’s accuracy while reducing redundancy. Comparatively, features like fractal dimension mean and concavity worst demonstrated limited diagnostic value.

As shown in Table 4, previous studies have demonstrated the effectiveness of machine learning and deep learning approaches for breast cancer classification. However, many studies were limited by small sample sizes, lack of external validation, high computational requirements, reduced model interpretability, or dependence on large datasets. The present study achieved a classification accuracy of 98.68% and an AUC of 0.96 using the SVC-RBF classifier on the Wisconsin Breast Cancer Diagnostic dataset. The findings indicate that a comparatively simple machine learning framework combined with robust preprocessing and feature selection can achieve competitive performance while maintaining computational efficiency.

**Table 4.** Summary of related studies comparing datasets, machine learning models, performance outcomes, and limitations in breast cancer classification.

Reference	Dataset	Feature Extraction	Model	Results	Weaknesses
Tahmooresi et al. (2019) ^25^	Wisconsin Breast Cancer Dataset	Morphological and statistical features	SVM	Accuracy: 94%	Limited feature optimization; lack of external validation
Kayode et al. (2019) ^26^	Mammography Images	Image texture features	Modified SVM	Sensitivity: 94.4%; Specificity: 91.3%	Small dataset; limited generalizability
Shen et al. (2019) ^27^	CBIS-DDSM, INbreast	Automatic deep feature extraction	Deep CNN	AUC: 0.91–0.98	High computational requirements; reduced interpretability
Suh et al. (2020) ^28^	Digital Mammography Dataset	Deep feature extraction	DenseNet-169, EfficientNet-B5	AUC: 0.952–0.954	Requires large datasets and GPUs
Viswanath et al. (2019) ^29^	Mammography Dataset	Image processing features	Random Forest	Accuracy: 84.84%	Lower predictive performance
Hussain et al. (2024) ^30^	Multiple datasets	Not applicable	Systematic Review	Comprehensive review	No experimental validation
Present Study	Wisconsin Breast Cancer Diagnostic Dataset	Robust Scaling + EDA-based Feature Selection	Logistic Regression, SVC, Decision Tree, Random Forest	SVC-RBF Accuracy: 98.68%; AUC: 0.96	Single dataset; no external validation

The findings surpass prior studies in terms of model performance. For instance, M. Tahmooresi et al. ^25^ reported an SVM accuracy of 94%, while Shen et al., ^27^ developed a deep learning algorithm for breast cancer detection on mammograms using an “end-to-end” approach, achieving high accuracy across heterogeneous datasets such as CBIS-DDSM (AUC: 0.91) and IN breast (AUC: 0.98). This improvement is attributed to advanced preprocessing techniques, such as robust scaling and hyperparameter tuning, combined with a comprehensive evaluation framework. Kayode et al.’s ^26^ SVM model achieved a sensitivity of 94.4% and specificity of 91.3%, and Debelee et al. ^31^ reported 99% accuracy on the BGH dataset. While these results are comparable, this study’s comprehensive evaluation, including confusion matrix-derived metrics, adds rigor to the findings. Similarly, Suh et al. ^28^ explored neural network models, such as DenseNet-169 and EfficientNet-B5, achieving AUCs of 0.952–0.954. However, these models require larger datasets and computational resources, unlike the efficient SVC-RBF model used here. Notably, Viswanath et al.’s ^29^ Random Forest model showed balanced performance (accuracy 84.84%, precision 90%, specificity 89%), yet it underperformed compared to the SVC-RBF model in this study, emphasizing the latter’s ability to capture non-linear relationships in high-dimensional datasets.

Hussain et al. (2024) ^30^ provide a comprehensive review of machine learning models for breast cancer risk prediction, analyzing key algorithms such as deep learning, decision trees, support vector machines, and ensemble learning. Their study highlights the significance of dataset selection, feature engineering, and model interpretability in improving predictive accuracy. While their work offers a broad overview of machine learning in cancer diagnostics, our study focuses specifically on the Support Vector Classifier with an RBF kernel (SVC-RBF), evaluating its robustness and optimization for cancer classification. Additionally, while Hussain et al. discuss challenges such as dataset bias and feature selection, we extend this discussion by assessing kernel-based optimization and hyperparameter tuning, which play a crucial role in improving predictive performance in imaging-based diagnostics. Similarly, Uthamacumaran et al. (2023) ^32^ introduce a novel machine intelligence-driven classification approach for extracellular vesicles derived from cancer patients using fluorescence correlation spectroscopy (FCS). Their study emphasizes the potential of machine learning in non-invasive cancer diagnostics by combining FCS data with deep learning models and advanced feature extraction techniques. While their work focuses on biomarker-based classification, our study applies SVC-RBF to imaging datasets, exploring its efficiency in structured imaging data rather than fluorescence-based biomarker detection. Additionally, while their research explores deep learning techniques, our work investigates the interpretability and efficacy of kernel-based supervised learning approaches in cancer classification.

The SVC-RBF model offers significant advantages. Its transparency, facilitated by interpretability techniques and visual tools, ensures trust among clinicians, enhancing its potential as a decision-support tool. While the model operates as a black-box algorithm with limited inherent interpretability, its strong predictive capability makes it a valuable candidate for decision-support applications in clinical settings. To enhance clinician trust and eventual translatability, future work will focus on integrating model-agnostic interpretability techniques, such as SHAP values or feature attribution methods, to improve transparency and support clinical decision-making. ^33, 34^ This study demonstrates the efficacy of machine learning techniques in the early detection and differential diagnosis of benign and malignant breast lesions, with the Support Vector Classifier using a Radial Basis Function (SVC-RBF) kernel emerging as the most accurate model. The additional statistical validation using five-fold cross-validation confirms that the observed classification performance was not the result of random variation. The relatively low standard deviation values obtained across all classifiers indicate consistent performance across multiple validation subsets. ^35– 37^ Furthermore, the low RMSE values observed for Logistic Regression, SVC Linear, and SVC-RBF demonstrate reliable prediction capability and robust model generalization. These findings provide further evidence supporting the reproducibility and clinical applicability of the proposed machine learning framework for breast cancer classification.

### 4.1 Strength and limitations of the study

A major strength of this study is the systematic evaluation and comparison of multiple machine learning algorithms for breast cancer classification using a standardized benchmark dataset. Comprehensive exploratory data analysis, robust data preprocessing, feature selection, hyperparameter tuning, and five-fold cross-validation were performed to improve model reliability and reduce bias. The SVC-RBF model demonstrated excellent diagnostic performance, achieving high accuracy, sensitivity, specificity, F1-score, and ROC-AUC values, highlighting its potential utility in supporting early breast cancer diagnosis.

However, several limitations should be acknowledged. The study relied on the Wisconsin Breast Cancer Diagnostic dataset, which is limited in size and diversity and may restrict the generalizability of the findings to broader clinical populations. External validation using independent or multicentre datasets was not performed. Although the SVC-RBF model achieved excellent predictive performance, its black-box nature limits interpretability. Advanced dimensionality reduction techniques such as PCA or t-SNE were not explored, and formal overfitting assessments, including learning curve or bias-variance analyses, were not conducted. In addition, feature selection was primarily based on correlation analysis and exploratory visualization and may not fully capture complex feature interactions. Statistical significance testing for feature-level separability was also not performed. Future studies should incorporate larger and more diverse datasets, external validation, advanced explainable AI methods such as SHAP, ensemble learning approaches, and multimodal data sources to further improve model robustness, interpretability, and clinical applicability.

## 5. Conclusion

This study developed and evaluated multiple machine learning classifiers for the classification of benign and malignant breast lesions using the Wisconsin Breast Cancer Diagnostic dataset. Among the evaluated models, the Support Vector Classifier with Radial Basis Function (SVC-RBF) achieved the highest classification performance, with an accuracy of 98.68% and an AUC of 0.96, demonstrating its effectiveness for breast cancer detection. The findings indicate that appropriate preprocessing, feature selection, and model optimization can substantially improve diagnostic performance and support early disease identification.

Despite these promising results, the study was limited by the use of a single publicly available dataset and the absence of external validation. Future studies should focus on validating the proposed framework using larger and more diverse multicentre datasets. The incorporation of multimodal imaging data, explainable artificial intelligence techniques, and advanced deep learning approaches may further improve model performance and facilitate clinical implementation.

## Ethics and consent

The dataset was extracted from the online open-source Wisconsin (Diagnostics) dataset. The study approval was obtained from Institutional Research Committee of Manipal College of Health Professions, Manipal on the 20 ^th^ of January 2022 (MCHP/Mpl/IRC/PG/2022/04). All procedures adhered to established ethical guidelines for secondary data analysis and data use policies. Consent is not applicable since the data was extracted from the online open source Wisconsin (Diagnostics) dataset.

## Data Availability

Kaggle: Wisconsin Breast Cancer Dataset, https://www.kaggle.com/datasets/uciml/breast-cancer-wisconsin-data . The dataset used in this study is publicly available from the Wisconsin Breast Cancer Diagnostic dataset on Kaggle, and the analysis code for data preprocessing, model development, hyperparameter tuning, and evaluation is available at: https://github.com/rinsyrahman/breast-cancer-ml-analysis. The data sets of mammography with benign and malignant breast lesions. Data are available under the terms of the CC BY-NC-SA 4.0 (CC-BY 4.0).
