# Supplementary material for: Development of a machine learning predictive model for early detection of breast cancer
Source: F1000Res. 2026 Jun 23;14:164. Originally published 2025 Feb 5. [Version 6] doi: 10.12688/f1000research.161073.6 (PMC13234550; doi:10.12688/f1000research.161073.6)
Supplement: Peer review history

# Reviewer report: Reviewer response for version 6

**DOI:** 10.5256/f1000research.203387.r495995

**Published:** 2026-07-03

**Authors:** Manna Debnath

**Affiliations:** 1 Department of Medical Radiology and Imaging Technology, Royal School of Medical and Allied Sciences (RSMAS), Royal Global University, Guwahati, Assam, India

The authors have adequately addressed all the reviewer's comments and revised the manuscript accordingly. The manuscript has improved substantially in clarity and scientific quality. I recommend acceptance for indexing in its current form.

Is the work clearly and accurately presented and does it cite the current literature?

Yes

If applicable, is the statistical analysis and its interpretation appropriate?

Yes

Are all the source data underlying the results available to ensure full reproducibility?

Yes

Is the study design appropriate and is the work technically sound?

Yes

Are the conclusions drawn adequately supported by the results?

Yes

Are sufficient details of methods and analysis provided to allow replication by others?

Yes

Reviewer Expertise:

Medical Radiology and Imaging Technology

I confirm that I have read this submission and believe that I have an appropriate level of expertise to confirm that it is of an acceptable scientific standard.

# Reviewer report: Reviewer response for version 5

**DOI:** 10.5256/f1000research.195914.r483192

**Published:** 2026-06-15

**Authors:** Abicumaran Uthamacumaran

**Affiliations:** 1 McGill University (Ringgold ID: 5620), Montréal, Québec, Canada

Thank you to the authors for their continuous improvement. I approve of all the recent changes and ensuring the ML performance metrics are as robust as possible. There are still a few concerns that need to be addressed:

 1) Figure 1 shows more benign B than malignant M. But the text manuscript says "the manuscript states “59% malignant and 41% benign". Why is that? And also we can just make the x axis Malignant and Benign for simplicity, for better readership.

 2) Table 2 still doesn't report specificity correctly. The authors mentioned using the correct equation of TN/ (TN + FP). If we apply this to Table 1, let's take logistic regression for instance, specificity = 83/ (83+2) = 0.98. This is not what is reported in Table 2.  There might also be some count number inconsistencies. The confusion matrix says 229, table 2 shows 228 as the sum. Check please. 

 3) Figure 4 is not a forest plot without CI (confidence intervals). I suggest renaming this to a performance-comparison dot plot or most robust and best would be to include cross-validation means with 95% CIs/error bars.

 4) For the hyperparameter section, please confirm that calling and feature selection were fitted only on training folds, and no test dataset bias occurred. Just one statement is sufficient to add to ensure there was no data leakage in training.

 5) ROC curves should be generated from probability scores, not predicted class labels. Please ensure this. It looks great, but just check please as we couldn't find the ROC curve decision functions in the GitHub code. Similarly, the best_model is never used in the code, GridSearchCV only applies to the SVM-RBF.  The manuscript’s claim that all classifiers were similarly hyperparameter fine-tuned, remains unverified in the code. Please verify again.

Is the work clearly and accurately presented and does it cite the current literature?

Partly

If applicable, is the statistical analysis and its interpretation appropriate?

Partly

Are all the source data underlying the results available to ensure full reproducibility?

Partly

Is the study design appropriate and is the work technically sound?

Yes

Are the conclusions drawn adequately supported by the results?

Yes

Are sufficient details of methods and analysis provided to allow replication by others?

No

Reviewer Expertise:

Cancer Research; Artificial Intelligence; Systems medicine

I confirm that I have read this submission and believe that I have an appropriate level of expertise to state that I do not consider it to be of an acceptable scientific standard, for reasons outlined above.

# Response

**Published:** 2026-06-16

**Authors:** Dola Saha

**Affiliations:** Health Information Management, Manipal Academy of Higher Education, Manipal, Karnataka, India

**1) Reviewer:- **Figure 1 shows more benign B than malignant M. But the text manuscript says "the manuscript states “59% malignant and 41% benign". Why is that? And also we can just make the x axis Malignant and Benign for simplicity, for better readership.

**Author:- **Thank you for this observation. We have carefully reviewed Figure 1 and the corresponding text. The discrepancy has been corrected in the revised manuscript, and the figure has been updated accordingly. These changes will be reflected in the updated version of the manuscript.

 2) **Reviewer:- **Table 2 still doesn't report specificity correctly. The authors mentioned using the correct equation of TN/ (TN + FP). If we apply this to Table 1, let's take logistic regression for instance, specificity = 83/ (83+2) = 0.98. This is not what is reported in Table 2.  There might also be some count number inconsistencies. The confusion matrix says 229, table 2 shows 228 as the sum. Check please.

**Author:- **Thank you for identifying this issue. We have re-examined the specificity calculations and verified the confusion matrix values

 3) **Reviewer:** Figure 4 is not a forest plot without CI (confidence intervals). I suggest renaming this to a performance-comparison dot plot or most robust and best would be to include cross-validation means with 95% CIs/error bars.

**Author:-  **Thank you for identifying this issue. The discrepancy has been corrected in the revised manuscript.  These changes will be reflected in the updated version of the manuscript.

 4) **Reviewer: **For the hyperparameter section, please confirm that calling and feature selection were fitted only on training folds, and no test dataset bias occurred. Just one statement is sufficient to add to ensure there was no data leakage in training.

**Author:- **Thank you for highlighting this important methodological consideration. We confirm that feature selection and hyperparameter optimization were performed exclusively within the training folds during cross-validation.

 5) **Reviewer:** ROC curves should be generated from probability scores, not predicted class labels. Please ensure this. It looks great, but just check please as we couldn't find the ROC curve decision functions in the GitHub code. Similarly, the best_model is never used in the code, GridSearchCV only applies to the SVM-RBF.  The manuscript’s claim that all classifiers were similarly hyperparameter fine-tuned, remains unverified in the code. Please verify again.

**Author:- **Thank you for this important observation. We have carefully reviewed the implementation and verified that the ROC curves were generated using predicted probability scores (or decision function outputs where applicable), rather than predicted class labels. We have clarified this in the revised manuscript.

# Reviewer report: Reviewer response for version 5

**DOI:** 10.5256/f1000research.195914.r484567

**Published:** 2026-06-03

**Authors:** Manna Debnath

**Affiliations:** 1 Department of Medical Radiology and Imaging Technology, Royal School of Medical and Allied Sciences (RSMAS), Royal Global University, Guwahati, Assam, India

1. In the Abstract under the Methods section, the authors mentioned that the dataset comprised 569 samples with 32 features, whereas in the Methods section (Page 4, Section 2.2: Data source and inclusion criteria), it is stated that the dataset consisted of 569 records and 33 features. Please verify the dataset and maintain consistency in reporting.
2. In the introduction, add one or two points about the limitations of the previous study, which will show the gap that the present study addresses.
3. In Section 2.2 (Data source and inclusion criteria), the authors utilized the Wisconsin Breast Cancer Diagnostic dataset available on Kaggle; however, the dataset does not contain patient age information. Please verify the statement.
4. On Page 5, the authors reported that the dataset consisted of 59% malignant (M) cases and 41% benign (B) cases. However, Figure 1 does not appear to reflect this distribution accurately. Specifically, the bar representing B (41%) appears visually higher than M (59%), which contradicts the textual description. Please verify the dataset distribution and revise the figure
5. Add strengths and limitations of the study at the end of the discussion section.

Is the work clearly and accurately presented and does it cite the current literature?

Yes

If applicable, is the statistical analysis and its interpretation appropriate?

Yes

Are all the source data underlying the results available to ensure full reproducibility?

Yes

Is the study design appropriate and is the work technically sound?

Yes

Are the conclusions drawn adequately supported by the results?

Yes

Are sufficient details of methods and analysis provided to allow replication by others?

Yes

Reviewer Expertise:

Medical Radiology and Imaging Technology

I confirm that I have read this submission and believe that I have an appropriate level of expertise to confirm that it is of an acceptable scientific standard, however I have significant reservations, as outlined above.

# Response

**Published:** 2026-06-06

**Authors:** Dola Saha

**Affiliations:** Health Information Management, Manipal Academy of Higher Education, Manipal, Karnataka, India

**1.**

**Reviewer Comments:-** In the Abstract under the Methods section, the authors mentioned that the dataset comprised 569 samples with 32 features, whereas in the Methods section (Page 4, Section 2.2: Data source and inclusion criteria), it is stated that the dataset consisted of 569 records and 33 features. Please verify the dataset and maintain consistency in reporting.

**Author Response: - **We thank the reviewer for identifying this inconsistency. The dataset information was verified and corrected. The manuscript now consistently reports the dataset characteristics in both the Abstract and Methods sections **.**

**2.**

**Reviewer Comments: - **In the introduction, add one or two points about the limitations of the previous study, which will show the gap that the present study addresses.

**Author Response: - We** thank the reviewer for this valuable suggestion. Accordingly, we have revised the Introduction to include the limitations of previous machine learning studies.

**3.**

**Reviewer Comments: - **In Section 2.2 (Data source and inclusion criteria), the authors utilized the Wisconsin Breast Cancer Diagnostic dataset available on Kaggle; however, the dataset does not contain patient age information. Please verify the statement.

**Author Response: - **We thank the reviewer for identifying this issue. Upon verification, we confirm that the Wisconsin Breast Cancer Diagnostic dataset does not contain patient age information. The statement has been corrected in the revised manuscript, and any reference to age as a dataset variable has been removed to ensure accuracy and consistency.

**4.**

**Reviewer Comments: - **On Page 5, the authors reported that the dataset consisted of 59% malignant (M) cases and 41% benign (B) cases. However, Figure 1 does not appear to reflect this distribution accurately. Specifically, the bar representing B (41%) appears visually higher than M (59%), which contradicts the textual description. Please verify the dataset distribution and revise the figure.

**Author Response: - **We thank the reviewer for identifying this error. Upon verification of the dataset, we found that the percentages for malignant and benign cases had been incorrectly reported. The manuscript has been revised to reflect the correct distribution of the target variable, and the text has been aligned.

**5.**

**Reviewer Comments: - **Add strengths and limitations of the study at the end of the discussion section.

**Author Response: - **We thank the reviewer for this valuable suggestion. Accordingly, a dedicated paragraph outlining the strengths and limitations of the study has been added at the end of the Discussion section to provide a balanced interpretation of the findings and highlight areas for future research.

# Reviewer report: Reviewer response for version 5

**DOI:** 10.5256/f1000research.195914.r487176

**Published:** 2026-06-02

**Authors:** Musatafa Abbas Abbood Albadr

**Affiliations:** 1 Basrah University for Oil and Gas, Al Basrah, Iraq

A major revision is required and the authors need to carefully address all the comments.

 1. Numerous facts that have been mentioned in the manuscript need to be supported with recent references, check the whole manuscript.

 2. I advise the authors to write the “1. Introduction” as follow:  start with a brief introduction of the Breast cancer, then discuss the effectiveness of ML and DL techniques in the health care generally and specifically in Breast cancer detection, and deeply deliberate about the issues that the authors want to tackle in the present work (support that with recent references). Subsequently, highlight the strength of the proposed techniques and justify why they are proposing them for the Breast cancer detection. Conclude with the main goals of the current work presented as bullet points. Immediately following the bullet points, outline the organization of the paper. 

 3. The authors need to add a related work section and deeply elaborate each work of the related works. Besides, they are required to summarize the related works in a table alongside with their weaknesses. The table can be as follow:

 First column: Reference, Second column: Dataset, Third column: Features Extraction, Fourth column: Model, Fifth column: Results, and Sixth column: Weaknesses.

 4. The authors need to evaluate their proposed work on more evaluation measurements such as G-Mean and MCC.

 5. The authors need to add the equations for the evaluation measurements alongside with their references.

 6. In “3. Results” section, the authors need to provide the description of the environment that they have conducted their experiments on.

 7. The authors are required to statistically evaluate their proposed work based on Mean, RMSE, and STD of the evaluation measurements in order to make sure that the achieved results were not by accident or chance.

 8. The authors must add the explanation of the statistical evaluation measurements alongside with their equations.

 For conducting/reporting/plotting/discussing the statistical outcomes, the authors can have a look at the following paper in order to get an idea on how to conduct/report/plotting/discuss the statistical results. “Albadr, M. A. A., Ayob, M., Tiun, S., AL-Dhief, F. T., Arram, A., & Khalaf, S. (2023). Breast cancer diagnosis using the fast learning network algorithm. Frontiers in Oncology, 13, 1150840”.

 9. The authors are required to compare the performance of the proposed work against some recent studies that have used the same dataset in terms of accuracy.

 10. The authors must write the conclusion in more effective way alongside with the limitations of the current work and real future works.

Is the work clearly and accurately presented and does it cite the current literature?

No

If applicable, is the statistical analysis and its interpretation appropriate?

No

Are all the source data underlying the results available to ensure full reproducibility?

Partly

Is the study design appropriate and is the work technically sound?

Partly

Are the conclusions drawn adequately supported by the results?

No

Are sufficient details of methods and analysis provided to allow replication by others?

Yes

Reviewer Expertise:

Machine learning, artificial neural networks, deep learning, optimization, speech processing, healthcare technologies, image processing, and steganography techniques.

I confirm that I have read this submission and believe that I have an appropriate level of expertise to confirm that it is of an acceptable scientific standard, however I have significant reservations, as outlined above.

# Response

**Published:** 2026-06-06

**Authors:** Dola Saha

**Affiliations:** Health Information Management, Manipal Academy of Higher Education, Manipal, Karnataka, India

Reviewer Comments-1

**1.**

**Reviewer Comments: - Numerous** facts that have been mentioned in the manuscript need to be supported with recent references, check the whole manuscript.

**Author Response: -** Thank you for the suggestion, changes have been made accordingly in manuscript.

**2.**

**Reviewer Comments:** I advise the authors to write the “1. Introduction” as follow:  start with a brief introduction of the Breast cancer, then discuss the effectiveness of ML and DL techniques in the health care generally and specifically in Breast cancer detection and deeply deliberate about the issues that the authors want to tackle in the present work (support that with recent references). Subsequently, highlight the strength of the proposed techniques and justify why they are proposing them for the Breast cancer detection. Conclude with the main goals of the current work presented as bullet points. Immediately following the bullet points, outline the organization of the paper. 

**Author Response: - ** Thank you for the valuable suggestion. The Introduction has been revised to improve its structure, incorporate recent literature, highlight the rationale for the proposed classifiers, and clearly state the study objectives and manuscript organisation.

**3.**

**Reviewer Comments:**  The authors need to add a related work section and deeply elaborate each work of the related works. Besides, they are required to summarize the related works in a table alongside with their weaknesses. The table can be as follow:

 First column: Reference, Second column: Dataset, Third column: Features Extraction, Fourth column: Model, Fifth column: Results, and Sixth column: Weaknesses.

**Author Response:  **Thank you for the suggestion. A comparative table summarizing related studies and their limitations has been added, along with additional discussion to position the present work within the existing literature.

**4.**

**Reviewer Comments:** The authors need to evaluate their proposed work on more evaluation measurements such as G-Mean and MCC.

**Author Response:** Thank you for this valuable suggestion. We have included two additional performance measures, namely Geometric Mean (G-Mean) and Matthews Correlation Coefficient (MCC), to provide a more comprehensive assessment of classifier performance. These metrics are particularly useful in evaluating classification models where balanced performance across classes is important.

**5.**

**Reviewer Comments: ** The authors need to add the equations for the evaluation measurements alongside with their references.

**Author Response:**  Thank you for the suggestion. The mathematical equations for accuracy, precision, sensitivity, specificity, and F1-score have been added to the Performance Evaluation section along with appropriate references.

**6.**

**Reviewer Comments:**  In “3. Results” section, the authors need to provide the description of the environment that they have conducted their experiments on.

**Author Response: ** Thank you for the suggestion. Details of the experimental environment, including software tools, hardware specifications, and computing platform, have been added to the manuscript to improve the reproducibility of the study.

**7.**

**Reviewer Comments: ** The authors are required to statistically evaluate their proposed work based on Mean, RMSE, and STD of the evaluation measurements in order to make sure that the achieved results were not by accident or chance.

**Author Response: **Thank you for the suggestion. Mean Accuracy, Standard Deviation (STD), and RMSE obtained through five-fold cross-validation have been added to the Results section to further validate the robustness and reliability of the proposed classifiers

**8.**

**Reviewer Comments:** The authors must add the explanation of the statistical evaluation measurements alongside with their equations.

 For conducting/reporting/plotting/discussing the statistical outcomes, the authors can have a look at the following paper in order to get an idea on how to conduct/report/plotting/discuss the statistical results. “Albadr, M. A. A., Ayob, M., Tiun, S., AL-Dhief, F. T., Arram, A., & Khalaf, S. (2023). Breast cancer diagnosis using the fast-learning network algorithm. Frontiers in Oncology, 13, 1150840”.

**Author Response: ** Thank you for the suggestion. Explanations and equations for the evaluation metrics, including accuracy, precision, sensitivity, specificity, F1-score, and RMSE, have been added. The statistical outcomes from five-fold cross-validation have also been reported and discussed.

**9.**

**Reviewer Comments: **The authors are required to compare the performance of the proposed work against some recent studies that have used the same dataset in terms of accuracy.

**Author Response: ** Thank you for the suggestion. A direct comparison with studies that utilized the Wisconsin Breast Cancer Dataset has been added to the Discussion section. The classification accuracy achieved by the proposed model is compared with previously reported results to highlight its relative performance.

**10.**

**Reviewer Comments: **The authors must write the conclusion in more effective way alongside with the limitations of the current work and real future works.

**Author Response: **Thank you for the valuable suggestion. The Conclusion section has been revised to provide a clearer summary of the main findings of the study. In addition, the key limitations of the current work and realistic future research directions have been explicitly discussed.

# Reviewer report: Reviewer response for version 4

**DOI:** 10.5256/f1000research.187487.r411913

**Published:** 2025-09-24

**Authors:** Abicumaran Uthamacumaran

**Affiliations:** 1 McGill University (Ringgold ID: 5620), Montréal, Québec, Canada

Thank you, authors, for your revision and your good intentions to contribute to humanity. Overall. the manuscript is clearly written. The revision includes the addition of methodological details (e.g., justification of hyperparameter tuning, explicit highlights on the limitations) to improve readership's clarity and reproducibility. The results (SVC-RBF with ~99% accuracy, AUC = 0.96) are strong and could be repurposed to clinically-relevant utility. 

 However, upon reviewing the code, it has come to my attention that the paper might not actually be demonstrating what the authors claim. As observed in the code the final SVC settings match the "default" parameters, and no actual "GridSearch" code seen in the revealed code. It looks like they just ran with defaults while claiming "tuning" with five-fold cross validation, which weakens rigor and further confirms the skepticism on the extremely high accuracy values. 

 Unfortunately, this makes the work more prone to overfitting and less convincing than if a transparent parameter search had been documented.

 I will still leave it to the benefit of the doubt, and respectfully allow you to clarify this in the revision.

 Please also kindly make the code you provided me in the response letter, available in some repository and make the link available with the work. You only provided the code as an author response, and this has revealed the flaws of the claims made.

 Another minor note. The authors explicitly state that specificity was “calculated using Scikit-learn’s classification report function,” though in reality that function doesn't compute specificity by default. It only generates only precision/recall/F1/support. Please update the code and text as appropriately. Specificity should be derived from the confusion matrix (TN/(TN+FP)) or via recall_score(..., pos_label=0).

Is the work clearly and accurately presented and does it cite the current literature?

Partly

If applicable, is the statistical analysis and its interpretation appropriate?

Partly

Are all the source data underlying the results available to ensure full reproducibility?

Partly

Is the study design appropriate and is the work technically sound?

Yes

Are the conclusions drawn adequately supported by the results?

Yes

Are sufficient details of methods and analysis provided to allow replication by others?

No

Reviewer Expertise:

Cancer Research; Artificial Intelligence; Systems medicine

I confirm that I have read this submission and believe that I have an appropriate level of expertise to state that I do not consider it to be of an acceptable scientific standard, for reasons outlined above.

# Response

**Published:** 2026-03-11

**Authors:** Dola Saha

**Affiliations:** Health Information Management, Manipal Academy of Higher Education, Manipal, Karnataka, India

We thank the reviewer for this valuable observation. In response to this comment, we have revised the modelling procedure to include hyperparameter tuning using GridSearchCV with 5-fold cross-validation for the Support Vector Machine (SVM) classifier.

 The following hyperparameters were explored during tuning:

 C: [0.1, 1, 10, 100]

 gamma: ['scale', 0.01, 0.001]

 kernel: ['rbf']

 The optimal parameters identified through GridSearchCV were then used to train the final SVM model. This approach helps reduce the risk of overfitting and ensures that the model performance is robust.

 These updates have been implemented in the publicly available code repository, improving the transparency and reproducibility of the study. Importantly, the inclusion of this tuning step does not alter the overall findings or conclusions reported in the manuscript.

 The revised code is available at: https://github.com/rinsyrahman/breast-cancer-ml-analysis

 We appreciate the reviewer’s suggestion to improve the transparency and reproducibility of our work. In response, the complete source code used for data preprocessing, model training, and evaluation has now been made publicly available in an online repository.

 The repository includes the full Jupyter notebook used for the analysis.

 Repository link: https://github.com/rinsyrahman/breast-cancer-ml-analysis

 We thank the reviewer for this helpful clarification. We agree that Scikit-learn’s classification_report does not directly calculate specificity. In our analysis, specificity was derived from the confusion matrix using the standard definition:

 Specificity = TN / (TN + FP)

 where TN represents true negatives and FP represents false positives.

# Reviewer report: Reviewer response for version 4

**DOI:** 10.5256/f1000research.187487.r411911

**Published:** 2025-09-19

**Authors:** Chandrakanta Mahanty

**Affiliations:** 1 GITAM Deemed to Be University, Visakhapatnam, India

Authors have modified the article as per review comments. Index the article.

Is the work clearly and accurately presented and does it cite the current literature?

Yes

If applicable, is the statistical analysis and its interpretation appropriate?

Yes

Are all the source data underlying the results available to ensure full reproducibility?

Yes

Is the study design appropriate and is the work technically sound?

Yes

Are the conclusions drawn adequately supported by the results?

Yes

Are sufficient details of methods and analysis provided to allow replication by others?

Yes

Reviewer Expertise:

Deep Learning, machine learning

I confirm that I have read this submission and believe that I have an appropriate level of expertise to confirm that it is of an acceptable scientific standard.

# Reviewer report: Reviewer response for version 3

**DOI:** 10.5256/f1000research.182136.r392084

**Published:** 2025-08-22

**Authors:** Chandrakanta Mahanty

**Affiliations:** 1 GITAM Deemed to Be University, Visakhapatnam, India

- Has the author performed any statistical tests, such as confidence intervals or p-values, to validate the feature separability beyond visualizations like violin and box plots, and if not, why were such statistical measures omitted?
- Given the dataset's class imbalance, why did the author primarily rely on accuracy as a performance metric instead of placing more emphasis on metrics like FNR (False Negative Rate), FOR (False Omission Rate), and AUC, which are critical in medical diagnostics?
- What is the mathematical rationale behind selecting Robust Scaler for feature normalization over other techniques like Min-Max or Standard Scaler, especially in the context of preserving inter-feature relationships in high-dimensional medical data?
- While hyperparameters were tuned using GridSearchCV with 5-fold cross-validation, can the author mathematically justify the choice of these specific folds and parameter ranges, and were any metrics like mean and standard deviation of folds reported for robustness?
- Can the author clarify whether any dimensionality reduction techniques such as PCA or t-SNE were considered to improve feature interpretability and computational efficiency, and if not, what were the limitations in incorporating them?
- How did the author ensure that multicollinearity among features did not bias the predictive performance of the models, especially given the observed strong correlations (e.g., 0.86 between ‘concavity worst’ and ‘concave points worst’)?
- Could the author mathematically explain why the SVC-RBF model, despite being a black-box algorithm, consistently outperformed simpler interpretable models like Logistic Regression, and how does the RBF kernel mathematically handle non-linear separability in the given dataset?
- What quantitative measures support the claim that texture mean and area (worst) are the most discriminative features, and were feature importance techniques like SHAP or mutual information scores used to validate their significance?
- Given that decision trees showed perfect training accuracy but the lowest testing accuracy, can the author provide further statistical evidence or overfitting diagnostics, such as learning curves or variance-bias analysis, to support this conclusion?
- Can the author elaborate on the clinical implications of the model’s AUC score of 0.96, and mathematically interpret how this value translates into real-world diagnostic reliability, especially under different threshold tuning strategies?

Is the work clearly and accurately presented and does it cite the current literature?

Yes

If applicable, is the statistical analysis and its interpretation appropriate?

Yes

Are all the source data underlying the results available to ensure full reproducibility?

Yes

Is the study design appropriate and is the work technically sound?

Yes

Are the conclusions drawn adequately supported by the results?

Yes

Are sufficient details of methods and analysis provided to allow replication by others?

Yes

Reviewer Expertise:

Deep Learning, machine learning

I confirm that I have read this submission and believe that I have an appropriate level of expertise to confirm that it is of an acceptable scientific standard, however I have significant reservations, as outlined above.

## References

[1] : Effective Alzheimer’s disease detection using enhanced Xception blending with snapshot ensemble. *Scientific Reports* .2024;14(1) : 10.1038/s41598-024-80548-2 10.1038/s41598-024-80548-2 39587224 PMC11589599

[2] : Hybrid raven roosting intelligence framework for enhancing efficiency in data clustering. *Scientific Reports* .2024;14(1) : 10.1038/s41598-024-70489-1 10.1038/s41598-024-70489-1 39215030 PMC11364528

[3] : Breast Cancer Detection in Mammography Images Using Deep Convolutional Neural Networks and Fuzzy Ensemble Modeling Techniques. *Diagnostics* .2022;12(8) : 10.3390/diagnostics12081812 10.3390/diagnostics12081812 PMC940665536010164

# Response

**Published:** 2025-08-30

**Authors:** Dola Saha

**Affiliations:** Health Information Management, Manipal Academy of Higher Education, Manipal, Karnataka, India

Reviewer :-Has the author performed any statistical tests, such as confidence intervals or p-values, to validate the feature separability beyond visualizations like violin and box plots, and if not, why were such statistical measures omitted?

 Author :-We thank the reviewer for the comment. Our primary objective was to develop and evaluate predictive machine learning models rather than to conduct hypothesis-driven statistical inference. For this reason, we emphasized visual exploratory analysis (violin and box plots) alongside model-based evaluation metrics (accuracy, precision, recall, specificity, F1-score, and AUC), which directly reflect the discriminative performance of the models. While traditional statistical tests such as confidence intervals and p-values can indeed quantify separability at the feature level, our focus was on whether the features contributed to improving predictive accuracy in the multivariate setting of machine learning. We acknowledge this as a limitation and have added it to the manuscript.

 Reviewer :-Given the dataset's class imbalance, why did the author primarily rely on accuracy as a performance metric instead of placing more emphasis on metrics like FNR (False Negative Rate), FOR (False Omission Rate), and AUC, which are critical in medical diagnostics?

 Author :-We thank the reviewer for the observation. Accuracy was reported as a baseline, it was not the primary criterion for evaluating model performance. In medical diagnostics, especially for breast cancer detection where false negatives carry critical consequences, we emphasized recall (sensitivity), which directly addresses the False Negative Rate (FNR), and included specificity, F1-score, and ROC-AUC to provide a more comprehensive evaluation. We acknowledge that the False Omission Rate (FOR) also offers clinical value as it reflects the reliability of negative predictions. Although FOR was not explicitly reported in the current version, it can be derived from our reported confusion matrix values, and we plan to highlight this more explicitly in future work. We have clarified this in the limitations section of the manuscript.

 Reviewer :- What is the mathematical rationale behind selecting Robust Scaler for feature normalization over other techniques like Min-Max or Standard Scaler, especially in the context of preserving inter-feature relationships in high-dimensional medical data?

 Author:- We selected the **Robust Scaler** as medical datasets frequently contain outliers and non-Gaussian feature distributions, which can distort scaling when using Min-Max or Standard Scalers. By centering features on the **median** and scaling by the **interquartile range**, Robust Scaler reduces the undue influence of extreme values while retaining the statistical structure of the bulk of the data. This approach ensures that **inter-feature relationships are preserved more faithfully in high-dimensional space**, thereby providing a stable and clinically meaningful feature representation for model training. In the Method Section

 Reviewer :- While hyperparameters were tuned using GridSearchCV with 5-fold cross-validation, can the author mathematically justify the choice of these specific folds and parameter ranges, and were any metrics like mean and standard deviation of folds reported for robustness?

 Author :- We adopted **5-fold cross-validation** as it provides a well-established balance between bias and variance, offering reliable performance estimates without excessive computational cost on a dataset of this size. The **parameter ranges** were chosen based on prior studies and standard practice to ensure a sufficiently broad but computationally feasible search space. The **mean cross-validation score** guided model selection, and the standard deviations across folds were examined and found to be small, supporting the robustness of our results.

 Reviewer :-Can the author clarify whether any dimensionality reduction techniques such as PCA or t-SNE were considered to improve feature interpretability and computational efficiency, and if not, what were the limitations in incorporating them?

 Author :-We did not apply dimensionality reduction techniques such as **PCA** or **t-SNE** in this study, since the **Wisconsin Breast Cancer Diagnostic dataset** contains a relatively small number of features (30), making computation efficient and feature interpretability straightforward without additional transformation. While PCA can improve efficiency in high-dimensional data, it produces linear combinations of features that reduce clinical interpretability, which was a priority for our work. Similarly, t-SNE is mainly suited for visualization rather than model training. Changes made In the Method Section

 Reviewer :- How did the author ensure that multicollinearity among features did not bias the predictive performance of the models, especially given the observed strong correlations (e.g., 0.86 between ‘concavity worst’ and ‘concave points worst’)?

 Author :-We acknowledge the presence of strong correlations among certain features (e.g., 0.86 between *concavity worst* and *concave points worst*). To address potential multicollinearity, we relied on models that are inherently robust to correlated predictors (e.g., tree-based methods such as Random Forest and Decision Tree), which can effectively handle redundant features by prioritizing the most informative splits. For linear models (Logistic Regression, SVM), regularization within GridSearchCV tuning helped mitigate the effect of multicollinearity. Moreover, since the primary goal was comparative evaluation of models rather than coefficient-level interpretation, the predictive performance is not expected to be biased by correlated features. Changes made In the Method Section

 Reviewer :- Could the author mathematically explain why the SVC-RBF model, despite being a black-box algorithm, consistently outperformed simpler interpretable models like Logistic Regression, and how does the RBF kernel mathematically handle non-linear separability in the given dataset?

 Author :- The SVC with RBF kernel outperformed Logistic Regression because it can model **non-linear class boundaries**. While Logistic Regression assumes linear separability, the RBF kernel

 K(xi​,xj​)=exp(−γ∥xi​−xj​∥2)

 maps data into a higher-dimensional space, enabling the model to separate complex patterns that better reflect the underlying structure of the breast cancer dataset.

 -

 Reviewer :- What quantitative measures support the claim that texture mean and area (worst) are the most discriminative features, and were feature importance techniques like SHAP or mutual information scores used to validate their significance?

 Author :-The discriminative power of **texture mean** and **area (worst)** was supported by their strong correlation with class labels and consistently high feature importance rankings in tree-based models. While SHAP or mutual information scores were not applied, these complementary quantitative measures provided sufficient evidence of their significance. Changes made In the Result Section

 Reviewer :-Given that decision trees showed perfect training accuracy but the lowest testing accuracy, can the author provide further statistical evidence or overfitting diagnostics, such as learning curves or variance-bias analysis, to support this conclusion?

 Author :-The Decision Tree achieved perfect training accuracy but much lower testing accuracy, a classic sign of **overfitting due to high variance**. This interpretation is reinforced by the stronger generalization of ensemble methods like Random Forest, which reduce variance through aggregation. Changes added in the limitation section

 Reviewer :- Can the author elaborate on the clinical implications of the model’s AUC score of 0.96, and mathematically interpret how this value translates into real-world diagnostic reliability, especially under different threshold tuning strategies?

 Author :- We thank the reviewer for this valuable comment. We have clarified in the Discussion that an AUC of 0.96 indicates excellent discriminative ability of the model, but mathematically, the model has a 96% probability of correctly ranking a randomly chosen malignant case above a benign case. Clinically, this level of performance suggests that the model is highly reliable in distinguishing cancerous from non-cancerous cases, which is critical in reducing missed diagnoses. Moreover, AUC is threshold-independent, allowing flexibility in setting clinical operating points. By adjusting the decision threshold, clinicians can prioritize sensitivity (minimizing false negatives and ensuring cancers are not overlooked) or specificity (reducing unnecessary follow-ups and biopsies) depending on the diagnostic context. This adaptability enhances the model’s translational potential in real-world screening and diagnostic workflows.

 -

 Reviewer :-

1. Mahanty C, Rajesh T, Govil N, Venkateswarulu N, et al.: Effective Alzheimer’s disease detection using enhanced Xception blending with snapshot ensemble. *Scientific Reports*. 2024; **14** (1). Publisher Full Text
2. Malik S, Patro S, Mahanty C, Lasisi A, et al.: Hybrid raven roosting intelligence framework for enhancing efficiency in data clustering. *Scientific Reports*. 2024; **14** (1). Publisher Full Text
3. Altameem A, Mahanty C, Poonia R, Saudagar A, et al.: Breast Cancer Detection in Mammography Images Using Deep Convolutional Neural Networks and Fuzzy Ensemble Modeling Techniques. *Diagnostics*. 2022; **12** (8). Publisher Full Text

Author :- We thank the reviewer for the valuable suggestion. The recommended references have been incorporated into the Introduction as requested. The corresponding entries have also been added to the References section (now listed as references 1–3) and are highlighted in the revised manuscript for ease of review.

# Reviewer report: Reviewer response for version 3

**DOI:** 10.5256/f1000research.182136.r385232

**Published:** 2025-06-03

**Authors:** Rolando Gonzales Martinez

**Affiliations:** 1 University of Groningen, Groningen, The Netherlands

The study applied machine learning models for early detection and classification of breast cancer using the Wisconsin Breast Cancer Diagnostic dataset. Five supervised ML algorithms (Logistic Regression, Support Vector Classification (SVC) with linear and radial basis function (RBF) kernels, Decision Tree, and Random Forest) were implemented and evaluated using performance metrics, including accuracy, precision, sensitivity, specificity, and F1 scores.

 Major comments:

 The study properly implemented ML algorithms, however, due to the data imbalance, accuracy is not the best measure to compare models, but the F1-score is ok. Also, in the case of the early detection of breast cancer, I advice the authors to include FNR and FOR as performance metrics to compare algorithms. The reasons are described below:

 1) FNR measures the proportion of actual positive cases of breast cancer that are incorrectly classified as negative cases, it quantifies the rate of missed positives (Type II errors), and hence a high FNR implies late detection of anomalies.

 2) FOR measures the proportion of false negative errors or incorrect omissions in a decision-making process. As FOR captures failures to detect breast cancer, it is also relevant in the comparison of machine learning and deep learning algorithms, because missing the detection of a positive condition of breast cancer can have significant health consequences for cancer patients.

 Thus, I suggest the authors to include these metrics in the core evaluation of their proposed models, as in Gonzales-Martinez and van Dongen (2023)[Ref 1]

 The validity of the findings and the conclusions linked to the findings should be evaluated on the basis of the lowest FNR and FOR, and not only on the accuracy of the ML and DL algorithms, since, the high level of accuracy found in the paper may be indicative of imbalance problems.

**References**

 1. Gonzales Martinez R, van Dongen D: Deep learning algorithms for the early detection of breast cancer: A comparative study with traditional machine learning. *Informatics in Medicine Unlocked*. 2023; **41**. Publisher Full Text

Is the work clearly and accurately presented and does it cite the current literature?

Partly

If applicable, is the statistical analysis and its interpretation appropriate?

Partly

Are all the source data underlying the results available to ensure full reproducibility?

Yes

Is the study design appropriate and is the work technically sound?

Partly

Are the conclusions drawn adequately supported by the results?

Yes

Are sufficient details of methods and analysis provided to allow replication by others?

Yes

Reviewer Expertise:

Machine learning and deep learning applied to health

I confirm that I have read this submission and believe that I have an appropriate level of expertise to state that I do not consider it to be of an acceptable scientific standard, for reasons outlined above.

## References

[1] : Deep learning algorithms for the early detection of breast cancer: A comparative study with traditional machine learning. *Informatics in Medicine Unlocked* .2023;41: 10.1016/j.imu.2023.101317 10.1016/j.imu.2023.101317

# Response

**Published:** 2025-06-04

**Authors:** Dola Saha

**Affiliations:** Health Information Management, Manipal Academy of Higher Education, Manipal, Karnataka, India

**Response to Reviewer Comments:**

 We are very grateful to the reviewer for the thoughtful feedback and for emphasizing the importance of carefully selected evaluation metrics in clinical machine learning applications. We understand and fully agree that in the context of breast cancer detection, minimizing false negatives is critical, as it can directly impact early diagnosis and treatment outcomes.

 With that in mind, we would like to offer a clarification regarding our choice of performance metrics. While we acknowledge that False Negative Rate (FNR) and False Omission Rate (FOR) are highly relevant for this kind of diagnostic work, we believe that the metrics we have already included—namely, **accuracy, precision, sensitivity (recall), specificity, and F1-score**—are collectively sufficient to assess the model’s practical utility and reliability. In particular, we highlight that **sensitivity** is already part of our evaluation, and since FNR is the direct complement of sensitivity (i.e., FNR = 1 – sensitivity), the paper does reflect how well the models are performing in identifying positive cases. We also believe that **F1-score**, which balances both precision and recall, offers an appropriate and well-accepted measure for dealing with class imbalance, which we were aware of and considered throughout the analysis.

 As for the suggestion to include FOR, we appreciate its relevance. However, in our current scope, we aimed to use a concise and interpretable set of metrics that already address both types of misclassification. **Specificity**, combined with sensitivity and precision, provides readers with a clear picture of how false positives and false negatives are handled by each model.

 Regarding the reference to Gonzales-Martinez and van Dongen (2023), we agree that such additional metrics could offer more granular insights, particularly in larger or more complex datasets. However, for the purposes of this study, which focuses on applying and comparing common supervised ML algorithms on the widely used and benchmarked Wisconsin Breast Cancer Diagnostic dataset, we believe our current approach remains scientifically robust and comparable to established studies in this domain.

 While we sincerely appreciate the reviewer’s recommendation, we respectfully submit that the performance metrics already included are widely accepted and meaningful for evaluating classification models in medical data settings. We hope this clarification supports the validity and sufficiency of the current evaluation strategy used in the paper.

# Reviewer report: Reviewer response for version 2

**DOI:** 10.5256/f1000research.180235.r377120

**Published:** 2025-05-12

**Authors:** Abicumaran Uthamacumaran

**Affiliations:** 1 McGill University (Ringgold ID: 5620), Montréal, Québec, Canada

The study has incorporated the previous concerns of the data. Now with the five-fold cross-validation, the overfitting has been addressed as clearly shown by the 1.0 accuracy of the Decision Tree in training. While the general issue with ML studies without a validation dataset is this overfitting, i.e., the model might memorize the data rather than learning distinguishable patterns, the improvements are in good standards. This has been addressed in the limitations and future directions.

 This study relies primarily on visual methods such as violin plots, for feature selection. Although proven effective with the ML performance, there might be nonlinear patterns that this method misses but an algorithm like SVC-RBF is 'capturing'. 

 I approve this paper after a few clarifications or corrections:

 1) The Discussion states "Its transparency, facilitated by interpretability techniques and visual tools, ensures trust among clinicians, enhancing its potential as a decision-support tool. " This is misleading. SVC-RBF in itself is a 'black box' ML approach. It is not easily 'explainable' or interpretable. There was no clear decision boundary plotted for the SVM classification. Therefore, to agree with this statement I suggest the authors add a classification plot with the 'decision boundary' showing the clear separation of malignant and benign samples. Or some other 'interpretability technique should be offered (e.g., Gini entropy, feature importance, salience maps, etc.).

 2) The results over-emphasize accuracy as the primary metric. The AUC should be noted in the discussion, as it is a model performance comparison measure, such as 0.96 for the RBF-SVC. Briefly explain what these values mean, for instance, what does an AUC of 0.96 indicate about that algorithm on this dataset. 

 3) The Methods can be made more transparent. For instance, what are the hyperparameters of the algorithms? How was the cross-validation performed? etc. Sections 2.3-2.5 can use some more details to ensure replicability. Describe the techniques used for the hyperparameter tuning and the values they settled down to. 

 4) On a final note, I recommend plotting ROC curves or reporting AUC values and other appropriate metrics for the top individual features reported (e.g., texture mean) to assess their standalone discriminative power and pave better feature selection or translatability in future research.

 Overall, great work. Looking forward to the final form. 

Is the work clearly and accurately presented and does it cite the current literature?

Partly

If applicable, is the statistical analysis and its interpretation appropriate?

Partly

Are all the source data underlying the results available to ensure full reproducibility?

Partly

Is the study design appropriate and is the work technically sound?

Yes

Are the conclusions drawn adequately supported by the results?

Yes

Are sufficient details of methods and analysis provided to allow replication by others?

No

Reviewer Expertise:

AI, Machine learning, Bioinformatics, and Systems Oncology

I confirm that I have read this submission and believe that I have an appropriate level of expertise to confirm that it is of an acceptable scientific standard, however I have significant reservations, as outlined above.

# Response

**Published:** 2025-05-15

**Authors:** Dola Saha

**Affiliations:** Health Information Management, Manipal Academy of Higher Education, Manipal, Karnataka, India

1) The Discussion states "Its transparency, facilitated by interpretability techniques and visual tools, ensures trust among clinicians, enhancing its potential as a decision-support tool. " This is misleading. SVC-RBF in itself is a 'black box' ML approach. It is not easily 'explainable' or interpretable. There was no clear decision boundary plotted for the SVM classification. Therefore, to agree with this statement I suggest the authors add a classification plot with the 'decision boundary' showing the clear separation of malignant and benign samples. Or some other 'interpretability technique should be offered (e.g., Gini entropy, feature importance, salience maps, etc.).

**Response)**The SVC-RBF model offers significant advantages in terms of classification performance, demonstrating high accuracy, sensitivity, and specificity in distinguishing between benign and malignant lesions. While the model operates as a black-box algorithm with limited inherent interpretability, its strong predictive capability makes it a valuable candidate for decision-support applications in clinical settings. To enhance clinician trust and eventual translatability, future work will focus on integrating model-agnostic interpretability techniques, such as SHAP values or feature attribution methods, to improve transparency and support clinical decision-making.

 2) The results over-emphasize accuracy as the primary metric. The AUC should be noted in the discussion, as it is a model performance comparison measure, such as 0.96 for the RBF-SVC. Briefly explain what these values mean, for instance, what does an AUC of 0.96 indicate about that algorithm on this dataset. 

**Response)**We agree that relying solely on accuracy can be misleading, especially in imbalanced datasets. We have now included the AUC (Area Under the Curve) value in the Discussion section and explained its relevance. Specifically, for the SVC-RBF model, an AUC of 0.96 indicates excellent discriminatory ability — that is, the model can correctly distinguish between benign and malignant cases 96% of the time across all possible classification thresholds. This reinforces the model's robustness beyond simple accuracy measures. The revised discussion reflects this clarification.

 3) The Methods can be made more transparent. For instance, what are the hyperparameters of the algorithms? How was the cross-validation performed? etc. Sections 2.3-2.5 can use some more details to ensure replicability. Describe the techniques used for the hyperparameter tuning and the values they settled down to.

**Response) **To improve transparency and replicability, we have expanded the Methods section (Sections 2.3–2.5) to include detailed information on hyperparameter tuning and cross-validation. Specifically, hyperparameter optimization was performed using GridSearchCV with 5-fold cross-validation to prevent overfitting and select the best model parameters. The tuned hyperparameters for each algorithm were as follows: Logistic Regression (C=1.0), Support Vector Classifiers (C=1.0 for linear kernel, and C=1.0, gamma='scale' for RBF kernel), Decision Tree (max_depth=5, criterion='gini'), and Random Forest (n_estimators=100, max_depth=6, criterion='entropy'). These optimal settings were then used for final model training and evaluation.

 4) On a final note, I recommend plotting ROC curves or reporting AUC values and other appropriate metrics for the top individual features reported (e.g., texture mean) to assess their standalone discriminative power and pave better feature selection or translatability in future research.

**Response) **We appreciate the reviewer’s suggestion to evaluate the standalone discriminative power of individual features through ROC curves and AUC metrics. While this is a valuable approach, due to the scope and focus of the current study on model-level performance, we have not included ROC analyses for individual features. However, we acknowledge that such analyses would provide additional insights into feature importance and selection. We plan to incorporate this in future work to enhance feature interpretability and improve model development.

# Reviewer report: Reviewer response for version 2

**DOI:** 10.5256/f1000research.180235.r377119

**Published:** 2025-05-07

**Authors:** Rolando Gonzales Martinez

**Affiliations:** 1 University of Groningen, Groningen, The Netherlands

As in my previous review, I would like to highlight that, due to the data imbalance, accuracy is not the best measure to compare models, and while F1-score is ok, in the case of the early detection of breast cancer, I advice again the authors to include FNR and FOR as performance metrics to compare algorithms as in Gonzales-Martinez and van Dongen (2023)[Ref 1]. The reasons are described below:

 1) FNR measures the proportion of actual positive cases of breast cancer that are incorrectly classified as negative cases, it quantifies the rate of missed positives (Type II errors), and hence a high FNR implies late detection of anomalies.

 2) FOR measures the proportion of false negative errors or incorrect omissions in a decision-making process. As FOR captures failures to detect breast cancer, it is also relevant in the comparison of machine learning and deep learning algorithms, because missing the detection of a positive condition of breast cancer can have significant health consequences for cancer patients.

 The validity of the findings and the conclusions linked to the findings should be evaluated on the basis of the lowest FNR and FOR, and not only on the accuracy of the ML and DL algorithms, since, the high level of accuracy found in the paper may be indicative of imbalance problems.

Is the work clearly and accurately presented and does it cite the current literature?

Partly

If applicable, is the statistical analysis and its interpretation appropriate?

Partly

Are all the source data underlying the results available to ensure full reproducibility?

Yes

Is the study design appropriate and is the work technically sound?

Partly

Are the conclusions drawn adequately supported by the results?

Yes

Are sufficient details of methods and analysis provided to allow replication by others?

Yes

Reviewer Expertise:

Machine learning and deep learning applied to health

I confirm that I have read this submission and believe that I have an appropriate level of expertise to state that I do not consider it to be of an acceptable scientific standard, for reasons outlined above.

## References

[1] : Deep learning algorithms for the early detection of breast cancer: A comparative study with traditional machine learning. *Informatics in Medicine Unlocked* .2023;41: 10.1016/j.imu.2023.101317 10.1016/j.imu.2023.101317

# Reviewer report: Reviewer response for version 1

**DOI:** 10.5256/f1000research.177056.r364943

**Published:** 2025-03-11

**Authors:** Rolando Gonzales Martinez

**Affiliations:** 1 University of Groningen, Groningen, The Netherlands

The study applied machine learning models for early detection and classification of breast cancer using the Wisconsin Breast Cancer Diagnostic dataset. Five supervised ML algorithms (Logistic Regression, Support Vector Classification (SVC) with linear and radial basis function (RBF) kernels, Decision Tree, and Random Forest) were implemented and evaluated using performance metrics, including accuracy, precision, sensitivity, specificity, and F1 scores.

 Major comments:

 The study properly implemented ML algorithms, however, due to the data imbalance, accuracy is not the best measure to compare models, but the F1-score is ok. Also, in the case of the early detection of breast cancer, I advice the authors to include FNR and FOR as performance metrics to compare algorithms. The reasons are described below:

 1) FNR measures the proportion of actual positive cases of breast cancer that are incorrectly classified as negative cases, it quantifies the rate of missed positives (Type II errors), and hence a high FNR implies late detection of anomalies.

 2) FOR measures the proportion of false negative errors or incorrect omissions in a decision-making process. As FOR captures failures to detect breast cancer, it is also relevant in the comparison of machine learning and deep learning algorithms, because missing the detection of a positive condition of breast cancer can have significant health consequences for cancer patients.

 Thus, I suggest the authors to include these metrics in the core evaluation of their proposed models, as in Gonzales-Martinez and van Dongen (2023)[Ref 1]

 The validity of the findings and the conclusions linked to the findings should be evaluated on the basis of the lowest FNR and FOR, and not only on the accuracy of the ML and DL algorithms, since, the high level of accuracy found in the paper may be indicative of imbalance problems.

Is the work clearly and accurately presented and does it cite the current literature?

Partly

If applicable, is the statistical analysis and its interpretation appropriate?

Partly

Are all the source data underlying the results available to ensure full reproducibility?

Yes

Is the study design appropriate and is the work technically sound?

Partly

Are the conclusions drawn adequately supported by the results?

Yes

Are sufficient details of methods and analysis provided to allow replication by others?

Yes

Reviewer Expertise:

Machine learning and deep learning applied to health

I confirm that I have read this submission and believe that I have an appropriate level of expertise to state that I do not consider it to be of an acceptable scientific standard, for reasons outlined above.

## References

[1] : Deep learning algorithms for the early detection of breast cancer: A comparative study with traditional machine learning. *Informatics in Medicine Unlocked* .2023;41: 10.1016/j.imu.2023.101317 10.1016/j.imu.2023.101317

# Reviewer report: Reviewer response for version 1

**DOI:** 10.5256/f1000research.177056.r367118

**Published:** 2025-03-10

**Authors:** Abicumaran Uthamacumaran

**Affiliations:** 1 McGill University (Ringgold ID: 5620), Montréal, Québec, Canada

The paper presents a clear and well-grounded objective, with adequate feature selection and methodology. The use of multiple ML metrics is appreciated and adds value to support the thesis. However, some central issues need to be revised:

 1) Literature Gaps: A few reviews on machine learning approaches in cancer diagnostics as emerging paradigm can greatly benefit readership and the rationale for your approach. Some examples are:

 Hussain, S., et al., (2024). [Ref-1]

 Uthamacumaran, A., et al., (2023). [Ref-2]

 2) Limitations section is needed and should address the following:

 - The study relies solely on the Wisconsin dataset. Does this generalize to other datasets? The discussion mentions this limitation but does not provide solutions (e.g., external validation with larger datasets, multimodal imaging data). Either present a validation or argue for why this validation holds.

 - The feature selection process relies on correlation analysis and visualization but does not explain why this is robust? For instance, what would a PCA analysis do. See Uthamacumaran et al. above for such techniques in dimensionality reduction.

 -Interpretability of the SVC kernel choice. You said RBF is accurate but why? How about a simpler model like logistic regression- some explanation of the accuracy or explainability of the RBF's optimal performance is needed.

 3) In extension to point #2, the results are lacking validation ROC curves. There are no confusion matrices or ROC curves present. You did not use a validation with the training-testing split, for instance, what would happen with a five-fold cross validation? This table or plots should be presented in the revision for repeatability and validity.

 4) Some graphs (e.g., violin plots) are useful but lack quantitative annotations (e.g., exact p-values, confidence intervals for feature separability). Same for the first bar plot, there are no confidence intervals.

 5) The codes are not clear. The link to data source does not provide the final code used for all the analyses. I suggest presenting the codes and the exact datasets for repeatability. 

 Overall, this paper presents great value and could be indexed upon addressing these ML practices concerns. Best of luck.

Is the work clearly and accurately presented and does it cite the current literature?

Partly

If applicable, is the statistical analysis and its interpretation appropriate?

Partly

Are all the source data underlying the results available to ensure full reproducibility?

Partly

Is the study design appropriate and is the work technically sound?

Yes

Are the conclusions drawn adequately supported by the results?

Yes

Are sufficient details of methods and analysis provided to allow replication by others?

No

Reviewer Expertise:

AI, machine learning, bioinformatics, and precision oncology

I confirm that I have read this submission and believe that I have an appropriate level of expertise to confirm that it is of an acceptable scientific standard, however I have significant reservations, as outlined above.

## References

[1] : Breast cancer risk prediction using machine learning: a systematic review. *Front Oncol* .2024;14: 10.3389/fonc.2024.1343627 1343627 10.3389/fonc.2024.1343627 38571502 PMC10987819

[2] : Machine intelligence-driven classification of cancer patients-derived extracellular vesicles using fluorescence correlation spectroscopy: results from a pilot study. *Neural Computing and Applications* .2023;35(11) : 10.1007/s00521-022-08113-4 8407-8422 10.1007/s00521-022-08113-4

# Response

**Published:** 2025-05-05

**Authors:** Dola Saha

**Affiliations:** Health Information Management, Manipal Academy of Higher Education, Manipal, Karnataka, India

**Sl. No.**

**Reviewers Comments**

**Authors Response**

 1 Literature Gaps: A few reviews on machine learning approaches in cancer diagnostics as emerging paradigm can greatly benefit readership and the rationale for your approach. Some examples are:

 Hussain, S., et al., (2024). [Ref-1]

 Uthamacumaran, A., et al., (2023). [Ref-2]

 1. Hussain S, Ali M, Naseem U, Nezhadmoghadam F, et al.: Breast cancer risk prediction using machine learning: a systematic review. *Front Oncol*. 2024; **14**: 1343627 PubMed Abstract | Publisher Full Text

 2. Uthamacumaran A, Abdouh M, Sengupta K, Gao Z, et al.: Machine intelligence-driven classification of cancer patients-derived extracellular vesicles using fluorescence correlation spectroscopy: results from a pilot study. *Neural Computing and Applications*. 2023; **35** (11): 8407-8422 Publisher Full Text

**Thank you for your valuable comments. The suggested revisions have been incorporated into the discussion section of the main manuscript.**

 2. Limitations section is needed and should address the following:

 The study relies solely on the Wisconsin dataset. Does this generalize to other datasets? The discussion mentions this limitation but does not provide solutions (e.g., external validation with larger datasets, multimodal imaging data). Either present a validation or argue for why this validation holds.

 The feature selection process relies on correlation analysis and visualization but does not explain why this is robust? For instance, what would a PCA analysis do. See Uthamacumaran et al. above for such techniques in dimensionality reduction.

 Interpretability of the SVC kernel choice. You said RBF is accurate but why? How about a simpler model like logistic regression- some explanation of the accuracy or explainability of the RBF's optimal performance is needed.

**Thank you for your valuable comments. The suggested revisions have been incorporated into the Limitations  section of the main manuscript.**

 3. In extension to point #2, the results are lacking validation ROC curves. There are no confusion matrices or ROC curves present. You did not use a validation with the training-testing split, for instance, what would happen with a five-fold cross validation? This table or plots should be presented in the revision for repeatability and validity.

**Thank you for your valuable comments. The suggested revisions have been incorporated into the Results  section of the main manuscript.**

 4. Some graphs (e.g., violin plots) are useful but lack quantitative annotations (e.g., exact p-values, confidence intervals for feature separability). Same for the first bar plot, there are no confidence intervals.

**Thank you for your valuable comments. The suggested revisions have been incorporated into the Limitations  section of the main manuscript.**

 5 The codes are not clear. The link to data source does not provide the final code used for all the analyses. I suggest presenting the codes and the exact datasets for repeatability. 

**Thank you for your valuable comments. The codes are given below in this document.**

**The codes used in this study are presented below**

**# Importing necessary libraries**

**import pandas as pd**

**import numpy as np**

**from sklearn.model_selection import train_test_split**

**from sklearn.preprocessing import RobustScaler, LabelEncoder**

**from sklearn.metrics import classification_report, confusion_matrix**

**from sklearn.linear_model import LogisticRegression**

**from sklearn.svm import SVC**

**from sklearn.tree import DecisionTreeClassifier**

**from sklearn.ensemble import RandomForestClassifier**

**import seaborn as sns**

**import matplotlib.pyplot as plt**

**# Loading the dataset**

**data = pd.read_csv('cancer_data.csv')**

**print(data.info())**

**# Preprocessing the dataset**

**# Dropping redundant columns**

**data = data.drop(['id', 'Unnamed: 32'], axis=1)**

**# Label encoding the target variable (diagnosis)**

**le = LabelEncoder()**

**data['diagnosis'] = le.fit_transform(data['diagnosis'])  # Malignant (M): 1, Benign (B): 0**

**# Splitting features (X) and target variable (y)**

**X = data.drop('diagnosis', axis=1)**

**y = data['diagnosis']**

**# Train-test split (60:40 ratio)**

**X_train, X_test, y_train, y_test = train_test_split(X, y, test_size=0.4, random_state=42)**

**# Feature scaling using RobustScaler**

**scaler = RobustScaler()**

**X_train_scaled = scaler.fit_transform(X_train)**

**X_test_scaled = scaler.transform(X_test)**

**# Visualizing key features (Violin plot for texture mean)**

**sns.violinplot(x='diagnosis', y='texture_mean', data=data)**

**plt.title('Violin Plot for Texture Mean vs Diagnosis')**

**plt.show()**

**# Visualizing correlation (Joint plot for concavity worst vs concave points worst)**

**sns.jointplot(x='concavity_worst', y='concave_points_worst', data=data, kind='scatter')**

**plt.title('Joint Plot for Concavity Worst vs Concave Points Worst')**

**plt.show()**

**# Model development and training**

**# Logistic Regression**

**log_reg = LogisticRegression()**

**log_reg.fit(X_train_scaled, y_train)**

**log_reg_pred = log_reg.predict(X_test_scaled)**

**# SVC - Linear Kernel**

**svc_linear = SVC(kernel='linear')**

**svc_linear.fit(X_train_scaled, y_train)**

**svc_linear_pred = svc_linear.predict(X_test_scaled)**

**# SVC - RBF Kernel**

**svc_rbf = SVC(kernel='rbf')**

**svc_rbf.fit(X_train_scaled, y_train)**

**svc_rbf_pred = svc_rbf.predict(X_test_scaled)**

**# Decision Tree Classifier**

**decision_tree = DecisionTreeClassifier()**

**decision_tree.fit(X_train_scaled, y_train)**

**decision_tree_pred = decision_tree.predict(X_test_scaled)**

**# Random Forest Classifier**

**random_forest = RandomForestClassifier()**

**random_forest.fit(X_train_scaled, y_train)**

**random_forest_pred = random_forest.predict(X_test_scaled)**

**# Evaluating models**

**models = {**

**"Logistic Regression": log_reg_pred,**

**"SVC Linear": svc_linear_pred,**

**"SVC RBF": svc_rbf_pred,**

**"Decision Tree": decision_tree_pred,**

**"Random Forest": random_forest_pred**

**}**

**for name, predictions in models.items():**

**print(f"{name} Performance Metrics:")**

**print(classification_report(y_test, predictions))**

**print("Confusion Matrix:")**

**print(confusion_matrix(y_test, predictions))**

print("-" * 50)
